# *Lacticaseibacillus rhamnosus* Glory LG12 preventives loperamide-induced constipation in mice by modulating intestinal flora and metabolic pathways

**DOI:** 10.3389/fmicb.2025.1577799

**Published:** 2025-07-11

**Authors:** Weiwei Ma, Lian Lian, Lidong Guo, Yanan Wu, Lili Huang

**Affiliations:** College of Pharmacy, Heilongjiang University of Chinese Medicine, Harbin, Heilongjiang, China

**Keywords:** *Lacticaseibacillus rhamnosus* Glory LG12, constipation, intestinal flora, short-chain fatty acids, metabolic pathway

## Abstract

**Introduction:**

Constipation is a common gastrointestinal disease, the incidence of which has been increasing year by year in recent years, and prolonged constipation seriously affects the physical and mental health of patients. Constipation often leads to dysbiosis of the intestinal flora, which in turn exacerbates intestinal dysfunction, and this may be an important mechanism for the development of constipation. Studies have shown that probiotics may be effective in relieving constipation by regulating intestinal flora. Among them, *Lacticaseibacillus rhamnosus* Glory LG12 (*L. rhamnosus* Glory LG12), as a potential probiotic strain, has attracted much attention in regulating intestinal flora and improving intestinal function. Although it has shown potential in the treatment of gastrointestinal disorders, its specific role in the treatment of constipation and the related mechanisms are unknown and require in-depth study.

**Methods:**

In the present study, a mouse model of constipation was constructed by loperamide hydrochlorid. The effect and mechanism of *L. rhamnosus* Glory LG12 on constipation were investigated by the indicators of water content of defecation, small intestine transit rate, time to the first black stool, defecation and number of grains in 5 h defecation, colonic pathology, inflammatory factors, neurotransmitters, short-chain fatty acids (SCFAs), intestinal flora and other indicators.

**Results:**

The results showed that *L. rhamnosus* Glory LG12 could prevent constipation symptoms to a great extent, and the preventive effect on constipation was more significant with the increasing dose of *L. rhamnosus*.

**Discussion:**

The mechanism of action may be related to the up-regulation of the abundance of *Bacteroidota*, *Firmicutes*, *Bacteroides*, *Ligilactobacillus* and *Parabacteroides* in the intestinal flora, the biosynthesis of amino acids, pyrimidine metabolism and other metabolic pathways, the promotion of a variety of glycoside hydrolases, and the increase of short-chain fatty acid content in the defecation of constipated mice.

## Graphical abstract

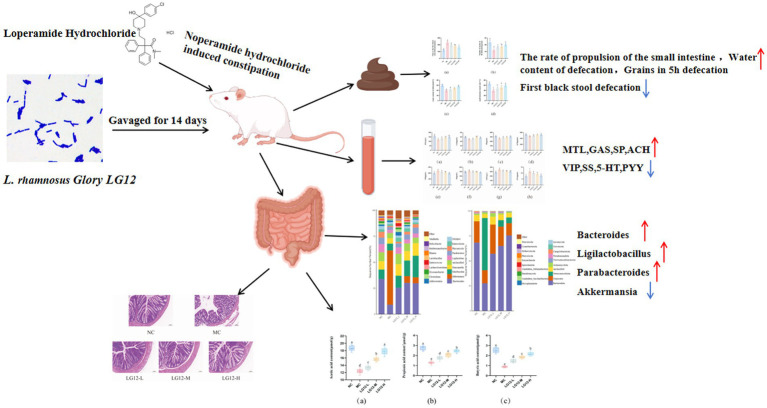

## 1 Introduction

Constipation is one of the common clinical gastrointestinal disorders, manifested by dry or lumpy defecation, reduced frequency of bowel movements, and incomplete or blocked defecation (Luan et al., 2019). Currently, the global prevalence of constipation is approximately 10–15 per cent and is increasing yearly (Imran et al., 2020). Chronic constipation symptoms are easy to recur, and the treatment effect is not good, so it brings both psychological and physical burden to the patient, seriously affecting the quality of life of the patient at the same time and also bringing a heavy burden of medical care for society (Xie et al., 2021). Studies have shown that long-term constipation can induce diseases such as haemorrhoids or even develop into bowel cancer, posing a serious threat to human health (Mody et al., 2012).

Therefore, effective measures need to be taken to prevent and treat constipation. Conventional treatments for constipation include diet, medication, surgery, etc. (Imran et al., 2020). Dietary therapy, which involves adjusting one’s diet and consuming more foods rich in dietary fiber, is not very effective. The main method of treating constipation with medication is to use laxatives, which stimulate intestinal peristalsis to promote defecation. However, long-term use can easily lead to dependence and cause damage to the body (Ford and Suares, 2011). In addition, drugs such as Prucalopride (Bassotti et al., 2019) and lubiprostone (Gras-Miralles and Cremonini, 2013) are also cause side effects such as headache, nausea, and abdominal pain (Liang et al., 2023b). Surgery is one of the least used treatments because of the potential risk of failure and complications, and is usually only used for patients with severe constipation (Knowles et al., 2017). There is an urgent need to develop new therapeutic strategies to overcome the limitations of existing approaches to treat constipation more safely and effectively. Probiotic preparations have gained widespread attention due to their high safety profile and ease of use compared to traditional drug therapy (Qiao et al., 2021). Probiotics are microorganisms that are beneficial to the host and can directly regulate the structure of intestinal flora and restore the microecological balance of the intestinal tract after moderate intake. Probiotics can increase the number of beneficial bacteria in the gut and inhibit the growth of harmful bacteria (Huang et al., 2024). In addition, probiotics have the ability to reduce intestinal inflammation, enhance the integrity of the intestinal mucosal barrier, and promote intestinal peristalsis, thus improving overall intestinal health and relieving constipation symptoms (Zheng et al., 2023b).

*Lacticaseibacillus rhamnosus* has been shown to regulate intestinal flora disorders and maintain intestinal microecology in mice (Mei et al., 2024). Han et al. (2024) administered *Lactobacillus rhamnosus* JYLR-127 (*L. rhamnosus* JYLR-127) to patients with constipation after bone fracture, and the results showed that the administration of *Lacticaseibacillus rhamnosus* was beneficial to the reconstruction of the destroyed intestinal flora and the modification of intestinal microecology. He et al. (2022) detected a significant increase in the abundance of *Lacticaseibacillus rhamnosus* in the intestinal tract of mice after stopping gavage of *Lactobacillus rhamnosus* LR22 (*L. rhamnosus* LR22) for 2 weeks, suggesting that *Lacticaseibacillus rhamnosus* colonised the intestinal tract of mice. The results showed that high-dose *Lacticaseibacillus rhamnosus* had better colonization effects than low-dose *Lacticaseibacillus rhamnosus*, thereby better regulating intestinal microbiota and relieving constipation. Wang G. et al. (2020) restored the relative abundance of Verrucomicrobia to normal levels in constipated mice after treatment with *Lactobacillus rhamnosus* CCFM 1068 (*L. rhamnosus* CCFM 1068), and they hypothesised that restoration of the abundance of Verrucomicrobia might be an important pathway for constipation relief by CCFM 1068. Borycka-Kiciak et al. (2017) administered the *Lacticaseibacillus rhamnosus* PL1 (*L. rhamnosus* PL1) strain for 9 weeks, the patients experienced a significant reduction in the intensity of pain, flatulence, abdominal discomfort and frequency of constipation. Available studies have shown that *Lacticaseibacillus rhamnosus* Glory LG12 (*L. rhamnosus* Glory LG12) can significantly increase the concentrations of lactic acid, acetic acid and butyric acid in the intestinal tract of mice (Ma et al., 2024). It also reduces intestinal permeability and strengthens the intestinal barrier (Zhang et al., 2024). The genome of *L. rhamnosus* Glory LG12 and GG, containing genes related to thermotolerance, oxygen tolerance, acid tolerance, bile salt tolerance and cold tolerance, were found to be identical in the previous study. In addition, *L. rhamnosus* Glory LG12 contains probiotic genes related to riboflavin, lactic acid, bioactive peptide synthesis, and related to immune regulation. Iprobiotics platform predicts that the likelihood of *L. rhamnosus* Glory LG12 being a probiotic is 99.89%. *L. rhamnosus* Glory LG12 did not detect genes associated with antibiotic resistance and did not contain any virulence genes (Details can be found in Annex 1). Based on previous studies and genomic predictions, it was shown that *L. rhamnosus* Glory LG12 is a safe strain of *L. rhamnosus* with high probiotic potential. The aim of this study was to investigate the preventive effect and mechanism of *L. rhamnosus* Glory *LG12* on a mouse model of loperamide hydrochloride- induced constipation, and provide more ideas for the development of products with the function of treating constipation, with the expectation of providing more choices for the treatment of constipation.

## 2 Materials and methods

### 2.1 Strain and materials

*L. rhamnosus* Glory *LG12* lyophilized powder (2 × 10^11^ CFU/g), Jinhua Galaxy Biotechnology Co. Ltd.; loperamide hydrochloride was purchased from MedChemexpress Biotechnology, United States; activated charcoal and gum arabic were purchased from Sinopharm Chemical Reagent Co. Ltd., China; motilin (MTL), gastrin (GAS), substance P (SP), acetylcholine (ACH), vasoactive intestinal peptide (VIP), Somatostatin (SS), Peptide YY (PYY), 5-hydroxytryptamine (5-HT); interleukin 1β (IL-1β), interleukin 6 (IL-6), interleukin 8 (IL-8), I interleukin 10 (L-10), Interferon-*γ* (IFN-γ), Tumor Necrosis Factor-*α* (TNF-α), the assay kit was purchased from Shanghai Enzyme-linked Biotechnology Co. Ltd., China.

### 2.2 *Lacticaseibacillus rhamnosus* Glory LG12 suspension preparation

According to China’s 《The Regulations on the Application and Evaluation of Probiotic Health Food》 stipulate that the number of live bacteria in probiotic health food during its shelf life should not be less than 10^6^ CFU/mL (g). In response to the above, 6.5 × 10^6^ CFU/mL was selected as the gavage dose for the low dose group, 0.2 mL per mouse by gavage. *L. rhamnosus* Glory LG12 was activated in MRS medium and incubated at 37°C for 24 h. The above procedure was then repeated for 2 more generations before the strain culture was used for the test. The culture solution was centrifuged at 5000 rpm for 10 min to collect the bacterial precipitate and washed twice with sterile saline. The concentration was then adjusted to that of the subsequent gavage: 6.5 × 10^6^ CFU/mL for the low-dose group, 6.5 × 10^7^ CFU/mL for the medium-dose group and 6.5 × 10^8^ CFU/mL for the high-dose group.

### 2.3 Animals and design of experiments

A total of 100 BALB/C male mice of 18–22 g were provided by Harbin Medical University. All animal operations were carried out in accordance with the Regulations on the Management of Laboratory Animals of Heilongjiang University of Chinese Medicine, and the experiments were approved by the Animal Ethics Committee of Heilongjiang University of Chinese Medicine (ethical approval code: 2024040701). One hundred mice were randomly divided into five groups: blank group (NC), model group (MC), low-dose group (LG12-L), medium-dose group (LG12-M), and high-dose group (LG12-H), with 20 mice in each group. The experiment was carried out in two groups of 10 animals each, A and B. Group A was used for the determination of small intestinal propulsion experiment and Group B was used for defecation experiment.

The animal feeding programme was based on the method of Ro et al. (2022) with brief modification. During the experimental period, all mice were fed food and water ad libitum and housed in IVC cages. The mice were fed with standard chow during the experimental period and housed in an environment with a temperature of 25 ± 2°C, relative humidity of 50 ± 5%, 12 h of light and 12 h of darkness. The animal experiments consisted of an adaptation period, an intervention period and a modelling period, totalling 22 days. Days 1–7 were the acclimatisation period, when mice were acclimatised for 1 week after being housed in the animal house. Days 8–21 were the intervention period, and 0.2 mL of sterile saline was gavaged every morning in the NC and MC groups, and 0.2 mL of 6.5 × 10^6^ CFU/mL,6.5 × 10^7^ CFU/mL, and 6.5 × 10^8^ CFU/mL of bacterial solution resuspended by sterile saline was gavaged in the LG12-L, LG12-M and LG12-H groups, respectively. On the 21 st day, the mice were gavaged in the afternoon and fasted without water for 16 h. Then on the 22 nd day, the NC group was gavaged with 0.2 mL of saline, and the other 4 groups were gavaged with 0.2 mL of loperamide hydrochloride (4 mg/kg B. W) to construct the mouse constipation model. After 0.5 h of gavage of loperamide hydrochloride, ink gavage was given to the NC and MC groups, and ink containing the corresponding test samples (ink:bacterial solution = 1:1) was given to the LG12-L, LG12-M and LG12-H groups, respectively, and the animals were immediately decapitated and put to death 25 min later.

### 2.4 Body weight measurement of mice

After the acclimatisation period, the body weights of the mice were examined every 7 days to observe the health status of the mice during the experimental period. The specific measurement method was as follows: each mouse was individually weighed before gavage to record the body weight of the mice. On the 22 nd day, the mice were weighed and killed by cervical dislocation method supplemented by blood sampling.

### 2.5 Determination of defecation index in mice

#### 2.5.1 Determination of the rate of propulsion of the small intestine

On the 22nd day of the experiment, 0.2 mL of physiological saline was gavaged in NC group A and 0.2 mL of loperamide hydrochloride (4 mg/kg B. W) was gavaged in the other 4 groups. After 0.5 h of gavage of loperamide hydrochloride, ink gavage was given to the NC and MC groups, and ink containing the corresponding subject samples was given to the low LG12-L, LG12-M, and LG12-H groups, respectively (ink:bacterial solution = 1:1). Immediately after 25 min, the animals were decapitated, the abdominal cavity was opened to separate the mesentery, and the intestinal tubes from the pylorus at the upper end and from the ileum at the lower end were clipped and placed on a tray, and the small intestines were gently pulled into a straight line, and the length of intestinal tubes was measured as the “total length of small intestines”, and that from the pylorus to the front of the ink was measured as the “length of the ink propulsion”. Calculate the small intestine transit rate according to the following formula:


$$\begin{array}{l} \mathrm{Small intestine transit rate}\;\left(\%\right)=\\ \;\;\;\;\;\;\;\;\;\;\;\;\;\frac{\mathrm{Ink}\;\mathrm{advance length}\;\left(\mathrm{cm}\right)}{\mathrm{Total length of small intestine}\;\left(\mathrm{cm}\right)}\times 100\% \end{array}$$

#### 2.5.2 Determination of water content of defecation

The method was the same as above, group B started from the infusion of ink, recorded the defecation water content of each mouse, collected the defecation pellets every 15 min and weighed them immediately, each mouse was collected 4 times and in the oven at 100°C, dried to constant weight, calculated the defecation water content of each group of mice. The water content of defecation was calculated according to the following formula:


$$\begin{array}{l} \mathrm{Water content of defecation}\;\left(\%\right)\;=\\ \;\;\;\;\frac{\mathrm{Fresh defecation weight}\;\left(g\right)-\mathrm{Dry}\;\mathrm{defecation weight}\;\left(g\right)}{\mathrm{Fresh defecation weight}\;\left(g\right)}\times 100\% \end{array}$$

#### 2.5.3 Determination of time to first black stool defecation and number of grains in 5 h defecation

The mice were housed in single cages with normal water and food, and the time of the first black stool and the number of black stools in 5 h were recorded for each mouse starting from ink infusion in group B.

### 2.6 Histopathological sections

The middle 0.7 cm of mouse colon was taken, preserved in fixative, embedded in paraffin overnight, and prepared for HE staining after paraffin sectioning of the colon tissue, and then the pathological changes of colon tissue were observed using a light microscope. The lesion analysis of the colon tissue was mainly based on the inflammatory cell infiltration, the arrangement of intestinal glands and the morphological structure of the colonic muscularis layer. Colonic histological damage criteria were scored according to Table 1.

**Table 1:** Colon histological injury standard score table.

Score	Degree of inflammatory cell infiltration	Alignment of intestinal glands
0	None	Neat
1	Mild	Mild disorder
2	Moderate	Moderate disorder
3	Serious	Serious disorder

### 2.7 Determination of serum neurotransmitters (excitatory/inhibitory)

The collected mouse blood was allowed to stand at room temperature for 1 h, centrifuged at 3500 × g for 15 min, and the supernatant was collected and set aside. The MTL, GAS, SP, ACH, VIP, SS, PYY and 5-HT levels were measured according to the instructions of the kit, and the concentrations of the substances to be measured in the samples were calculated from the absorbance.

### 2.8 Determination of inflammatory factors

The colonic tissue was gently rinsed using saline to remove any impurities remaining in the tissue. At the same time, the fat around the tissue was removed with surgical scissors and appropriate mass of mouse colon tissue was clipped. The tissues were weighed accurately, and pre-cooled sterile saline was added at the ratio of tissue mass:saline volume = 1:9 (w/v), and the tissues were crushed using a tissue crusher. The tissue was then centrifuged at 4°C and 3,500 r/min for 15 min, and the supernatant was collected. The levels of pro-inflammatory cytokines IL-1β, IL-6, IL-8, IFN-*γ*, TNF-*α* and anti-inflammatory cytokine IL-10 were determined in the mouse colon according to the method described in the ELISA kit.

### 2.9 Determination of short-chain fatty acid content

The mouse defecation collected before the end of the experiment were freeze-dried, and the dry weight of the defecation was weighed (about 20 mg), to which 500 μL of saturated NaCl solution was added for soaking and resuspension, and 40 μL of 10% sulphuric acid solution (v/v) was added along with 1,000 μL of anhydrous ether, and the fatty acids in the defecation were extracted by shaking well, and then centrifuged for 15 min using high-speed cryo-centrifuge with a speed of 12,000 r/min at 4°C. The upper ether phase was removed and 0.25 g of anhydrous sodium sulphate was added to remove the water. Then the fatty acids were extracted from the faeces by centrifugation at 12000 r/min in a high-speed freezer centrifuge at 4°C for 15 min. The upper ether phase was removed and 0.25 g of anhydrous sodium sulphate was added to remove the water. After standing for 30 min, the upper ether phase was also centrifuged at 12000 r/min 4°C for 5 min, and then the liquid was quickly transferred to a gas vial for measurement. The content of short-chain fatty acids (SCFAs) was determined by gas chromatography–mass spectrometry (GC–MS).

### 2.10 Metagenomic sequencing

DNA was extracted from colonic contents samples using CTAB method. DNA degradation degree, potential contamination and DNA concentration was measured using Agilent 5,400. Sequencing library was generated using NEBNext^®^ UltraTM DNA Library Prep Kit for Illumina (NEB, United States, Catalog#: E7370L) following manufacturer’s recommendations and index codes were added to each sample. Briefly, genomic DNA sample was fragmented by sonication to a size of 350 bp. Then DNA fragments were end polished, A-tailed, and ligated with the full-length adapter for Illumina sequencing, followed by further PCR amplification. After PCR products were purified by AMPure XP system (Beverly, United States). Subsequently, library quality was assessed on the Agilent 5,400 system (Agilent, United States) and quantified by QPCR (1.5 nM). The qualified libraries were pooled and sequenced on Illumina platforms with PE150 strategy, according to effective library concentration and data amount required.

### 2.11 Statistics and analysis of data

The data in this paper are presented as mean ± standard deviation (SD) for each group, and the experiments were conducted with at least 3 parallel groups, and the statistical analysis was done using GraphPad Prism 8 and Origin 8.5 software. The statistical methods used in which the data were analysed were one-way ANOVA with Dunnett’s post-hoc test, and the results were considered significant at *p* < 0.05.

## 3 Results

### 3.1 Effect of *Lacticaseibacillus rhamnosus* Glory LG12 on body weight in mice

The mice were treated by gavage of *L. rhamnosus* Glory LG12 for 14 d. Then loperamide was given on the last day to establish a constipation model, and the body weight of the mice changed during the experiment as shown in Table 2. During the experimental period, the body weight of mice in each group showed a trend of slow increase during probiotic gavage, there was no significant difference in the body weights of the mice on the first and last days (*p* > 0.05), indicating that the mice were in good physiological condition.

**Table 2:** Effect of *L. rhamnosus Glory LG12* on body weight of mice (*n* = 20).

Groups	Animals		Weight (g)	
		Day 8	Day 15	Day 22
NC	20	19.25 ± 0.71^a^	20.13 ± 0.76	21.29 ± 0.85^a^
MC	20	19.22 ± 0.67^a^	19.86 ± 0.77	21.31 ± 1.11^a^
LG12-L	20	19.56 ± 1.01^a^	20.72 ± 0.73	21.93 ± 0.68^a^
LG12-M	20	19.32 ± 1.08^a^	20.1 ± 1.07	21.76 ± 0.73^a^
LG12-H	20	19.35 ± 0.67^a^	20.62 ± 0.54	21.63 ± 0.78^a^

^aThe same letter indicate no significant difference.^

### 3.2 Effect of *Lacticaseibacillus rhamnosus* Glory LG12 on defecation indices in mice

**Figure 1:**
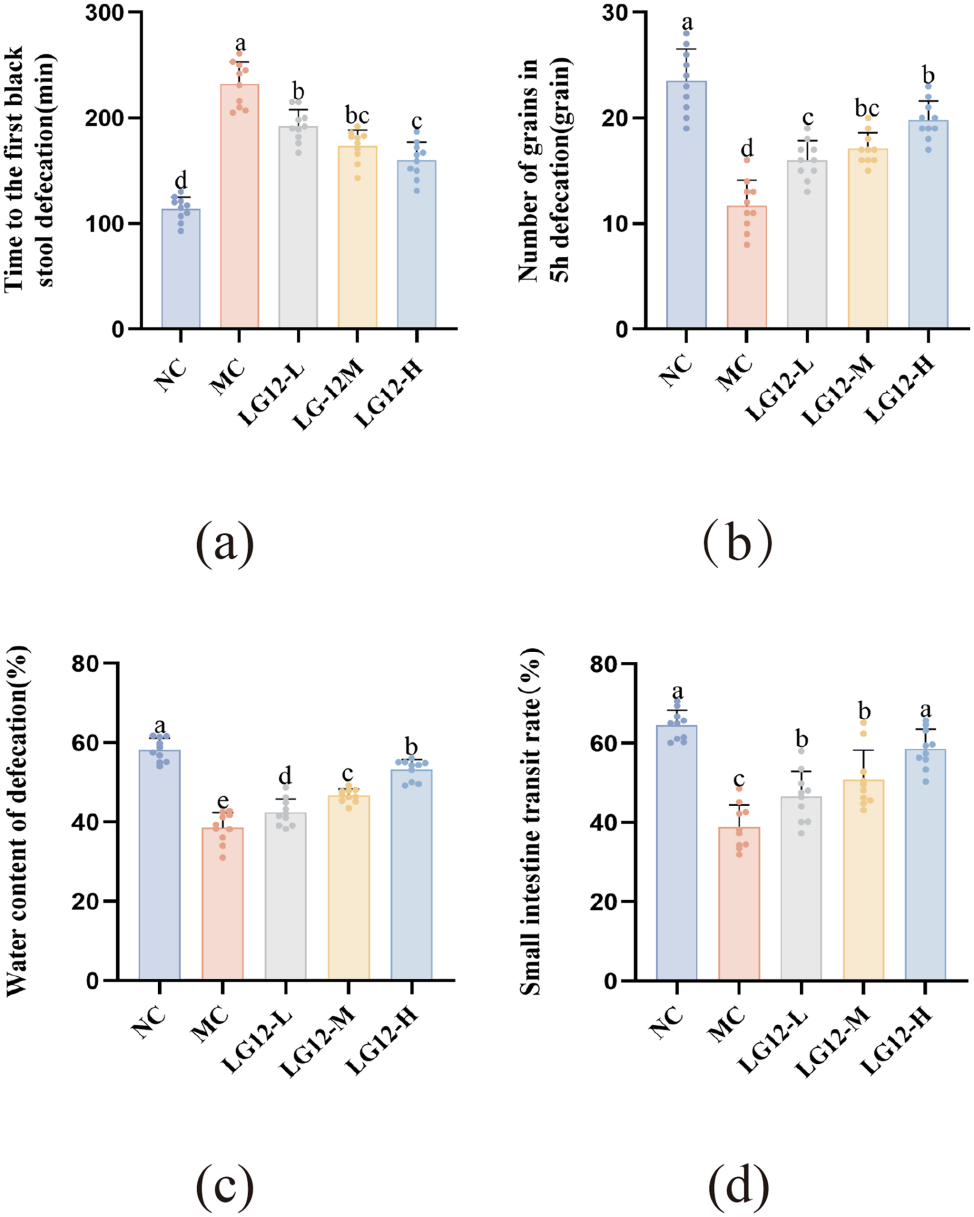
Effect of *L. rhamnosus Glory LG12* on defecation index in mice. **(a)** Time to the first black stool defecation; **(b)** Number of grains in 5 h defecation; **(c)** Water content of defecation; **(d)** Small intestine transit rate. a–e: Different letters indicate (*p* < 0.05), and the same letters or no letters indicate no significant difference (*n* = 10).

As can be seen from Figure 1a, the time to the first black stool of mice in the MC group was significantly prolonged compared with that of mice in the NC group (*p* < 0.05), suggesting that loperamide reduced the gastrointestinal motility, which laterally illustrated the success of the mouse constipation model. The time to first black stool was significantly shorter in the LG12-L, LG12-M and LG12-H groups than in the MC group (*p* < 0.05). The LG12-L, LG12-M and LG12-H groups reduced the time to black stool by 17.07, 25.17 and 30.99%. As can be seen from Figure 1b, in terms of the number of stool pellets in the 5 h bowel movement, the use of loperamide significantly reduced the number of stool pellets in the normal mice (*p* < 0.05), and it had an inhibitory effect on intestinal peristalsis. The number of faecal pellets was significantly increased 5 h after probiotic intervention compared to the MC group (*p* < 0.05). Among them, the LG12-L, LG12-M and LG12-H groups improved the number of 5 h bowel pellets by 36.75, 46.15 and 69.23%. From the results, it can be seen that the LG12-H group showed a significantly better response to the improvement of defecation indicators than the LG12-L group of *L. rhamnosus* Glory LG12, suggesting that *L. rhamnosus* Glory LG12 has a dose-dependent effect on constipation.

The results are shown in Figure 1c, which shows that the water content of mice defecation in the MC group was significantly lower (*p* < 0.05) compared with that of mice in the NC group. As can be seen from the figure, mice in the LG12-L, LG12-M, and LG12-H groups exhibited a significant increase (*p* < 0.05) in defecation water content compared to the MC group, and the LG12-L, LG12-M and LG12-H groups increased defecation water content by 10.12, 21.06, and 38.03%, respectively. The results show that *L. rhamnosus* Glory LG12 had a good effect on improving defecation water content in mice.

Loperamide hydrochloride triggers constipation by slowing down the peristalsis of the small intestine. Measurement of small intestinal propulsion rate provides insight into the effectiveness of *L. rhamnosus* Glory LG12 in preventing constipation. As shown in Figure 1d, the small intestinal propulsion rate of constipated mice was significantly lower (*p* < 0.05) in the MC group of mice compared to the NC group of mice. The small intestinal propulsion rate was significantly higher in the LG12-L, LG12-M and LG12-H groups than in the MC group (*p* < 0.05). The small intestinal propulsion rate of the LG12-H group was 50.90% higher than that of the MC group, which indicated that the small intestinal peristalsis of the constipated mice in the LG12-H group was well restored, and that *L. rhamnosus* Glory LG12 greatly preventive constipation in mice. In conclusion, the improved response to high-dose *L. rhamnosus* Glory LG12 treatment was more pronounced on defecation indices compared to low-dose and medium-dose *L. rhamnosus* Glory LG12, suggesting a dose-dependent effect of *L. rhamnosus* Glory LG12 on constipation.

### 3.3 Effect of *Lacticaseibacillus rhamnosus* Glory LG12 on pathological changes in mice colon tissue

**Figure 2:**
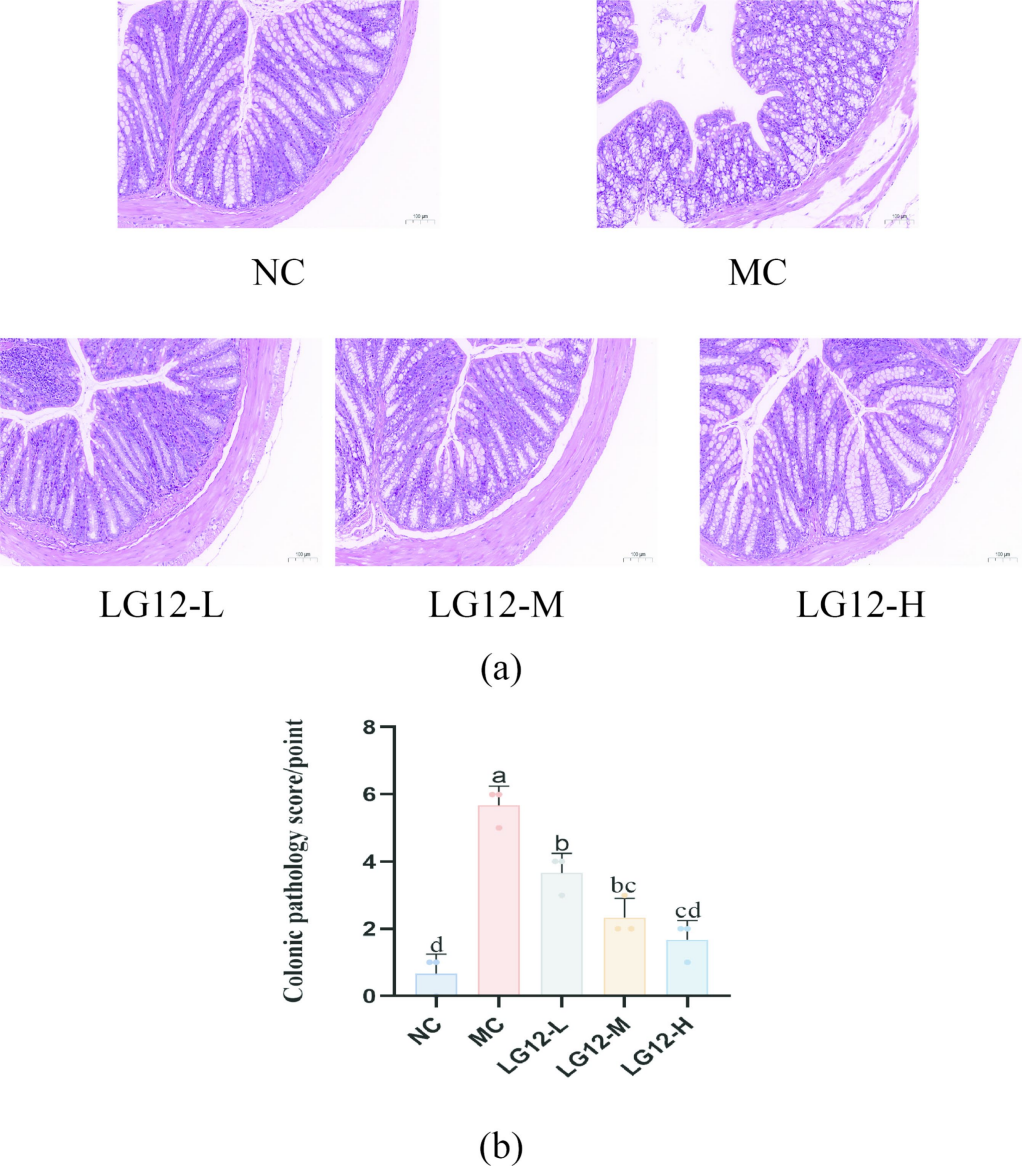
Effect of *L. rhamnosus Glory LG12* on pathological changes in mice colon tissues (original magnification ×200) and colonic pathology score. **(a)** Pathological changes in colon tissues, **(b)** Colonic pathology score. a–d: Different letters indicate (*p* < 0.05), and the same letters or no letters indicate no significant difference (*n* = 3).

As can be seen in Figure 2a, the intestinal glands in the mucosal layer of the colonic tissue in the NC group were neatly arranged and had a clear structure; as can be seen in Figure 2b, lymphocyte aggregation in the colonic tissue of the MC group was seen to be infiltrated by inflammatory cells, with disorganised intestinal glands and a thinning of the muscularis mucosae of the colon; As can be seen from Figure 2, the thickening of the colonic muscularis propria, the basic restoration of normal intestinal glandular arrangement, and the phenomenon of inflammatory infiltration were well improved in all dose groups after the intervention of *L. rhamnosus* Glory LG12. and showed a dose-dependency. This result was also verified by the colonic histological scores, which were significantly higher in the LG12-L group than in the LG12-H group (*p* < 0.05).

### 3.4 Effect of *Lacticaseibacillus rhamnosus* Glory LG12 on serum neurotransmitters in mice

**Figure 3:**
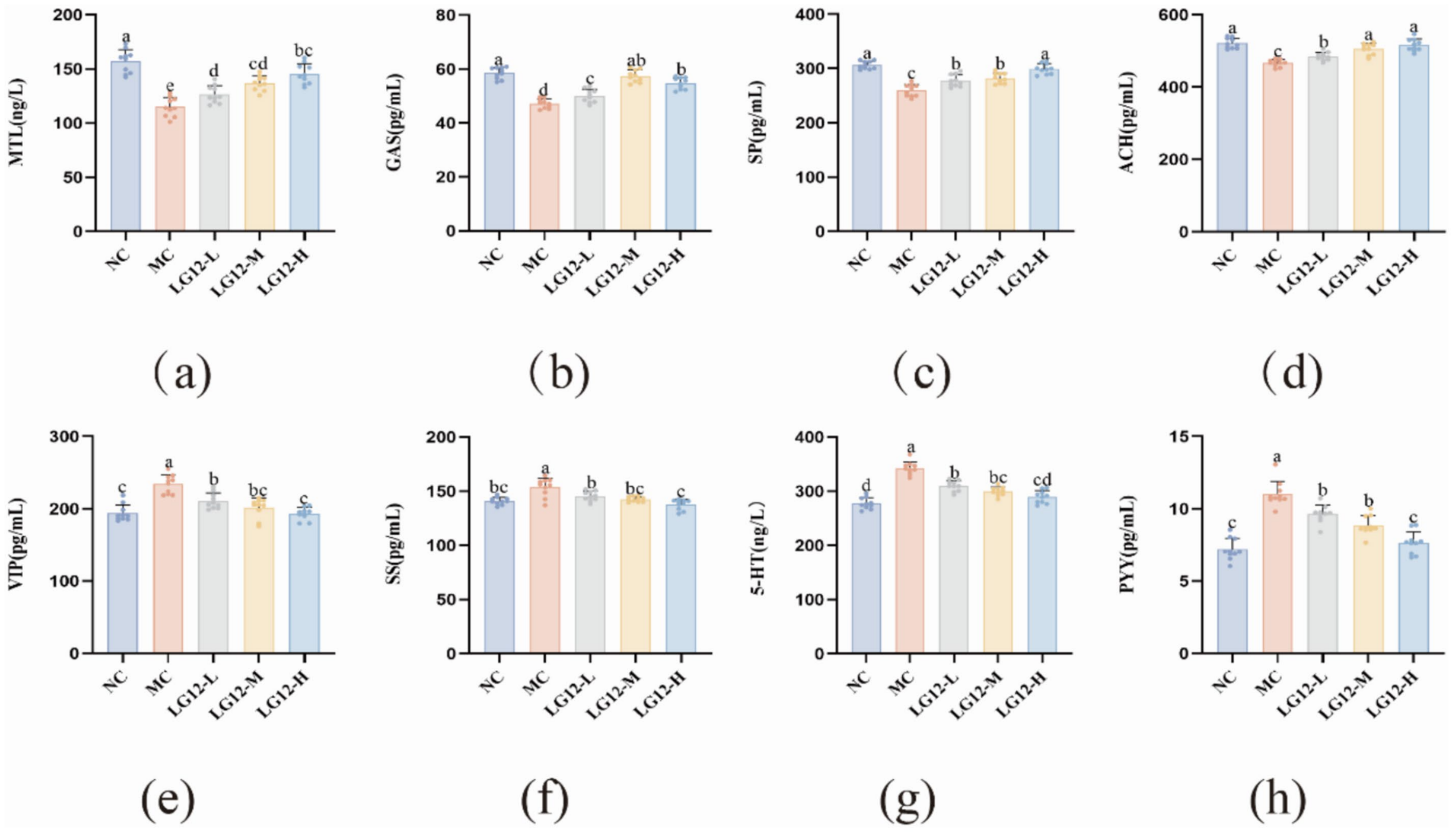
Effect of *L. rhamnosus Glory LG12* on serum neurotransmitters in mice. **(a)** MTL; **(b)** GAS; **(c)** SP; **(d)** ACH; **(e)** VIP; **(f)** SS; **(g)** 5-HT; **(h)** PYY. a–d: Different letters indicate (*p* < 0.05), and the same letters or no letters indicate no significant difference (*n* = 10).

Serum neurotransmitters can be broadly classified into excitatory and inhibitory types, and MTL, Gas, SP and ACH involved in this study belong to the excitatory type of transmitters, which play a contractile role in gastrointestinal smooth muscle, and can promote peristalsis of the gastrointestinal tract. Compared with the NC group, the content of excitatory transmitters in the MC group were all significantly decreased, and the differences were statistically different (*p* < 0.05). As can be seen in Figure 3, the levels of excitatory-type transmitters were significantly higher (*p* < 0.05) in mice in the LG12-L, LG12-M and LG12-H groups compared with the MC group. VIP, SS, PYY and 5-HT belong to the inhibitory-type transmitters, which inhibit the contraction of the small intestinal smooth muscle, which in turn leads to constipation. The experimental results showed that the levels of inhibitory transmitters were significantly increased in constipated mice compared with the NC group (*p* < 0.05). Compared with the MC group, the content of inhibitory transmitters was significantly decreased in the LG12-H group (*p* < 0.05). The results showed that *L. rhamnosus* Glory LG12 could promote the secretion of inhibitory transmitters such as MTL, Gas, SP, ACH etc., and then enhance the intestinal muscle motility of mice, which effectively improved the intestinal peristalsis of constipated mice.; Also, *L. rhamnosus* Glory LG12 reduces the concentrations of the inhibitory transmitters VIP, SS, PYY and 5-HT, and reduces the contraction of small intestinal smooth muscle, thereby treating constipation.

### 3.5 Effect of *Lacticaseibacillus rhamnosus* Glory LG12 on inflammatory factors in mice

**Figure 4:**
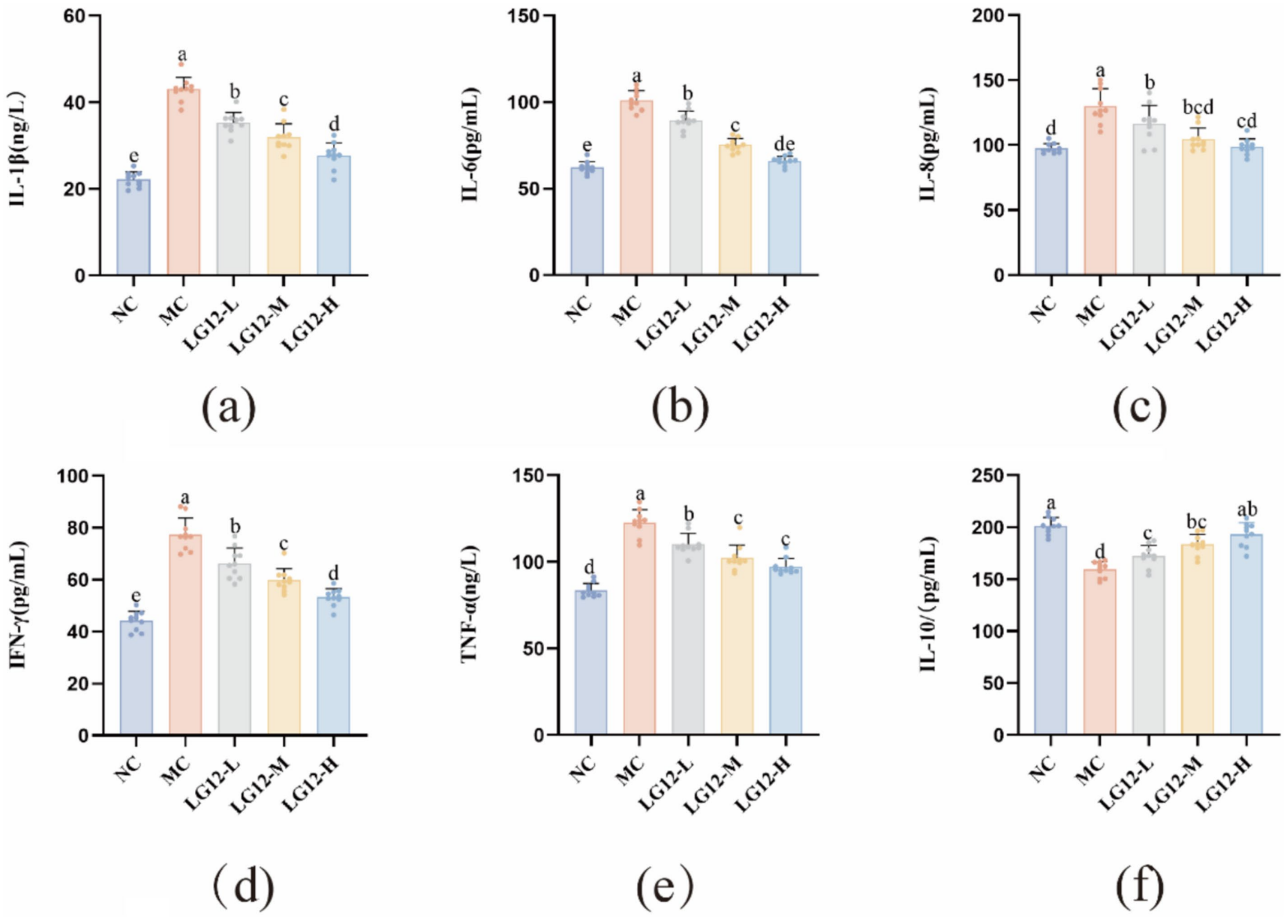
Effect of *L. rhamnosus Glory LG12* on intestinal cytokines in mice. **(a)** IL-1β; **(b)** IL-6; **(c)** IL-8; **(d)** IFN-γ; **(e)** TNF-*α*; **(f)** IL-10. a–e: Different letters indicate (*p* < 0.05), and the same letters or no letters indicate no significant difference (*n* = 10).

An important factor in the pathogenesis of constipation is the dysfunction of intestinal immunoregulation, and pro-inflammatory factors (IL-1β, IL-6, IL-8, IFN-*γ*, TNF-*α*) and anti-inflammatory factor IL-10 are key indicators of the degree of inflammation in the constipation model. As shown in Figure 4, the pro-inflammatory factors in MC group mice were significantly higher (*p* < 0.05) and the anti-inflammatory factors were significantly lower (*p* < 0.05) compared to NC group. Compared to the MC group, the content of anti-inflammatory factors in mice in the LG12-L, LG12-M and LG12-H groups was significantly increased (*p* < 0.05) after intervention treatment with *L. rhamnosus* Glory LG12. In addition, the content of pro-inflammatory factors in the intestines of mice in the LG12-L, LG12-M and LG12-H groups was significantly reduced after *L. rhamnosus* Glory LG12 intervention compared with the MC group (*p* < 0.05), indicating that *L. rhamnosus* Glory LG12 intervention could reduce the secretion of inflammatory cytokines and effectively prevent constipation in mice.

### 3.6 Effect of *Lacticaseibacillus rhamnosus* Glory LG12 on intestinal flora in mice

#### 3.6.1 Intestinal flora composition

**Figure 5:**
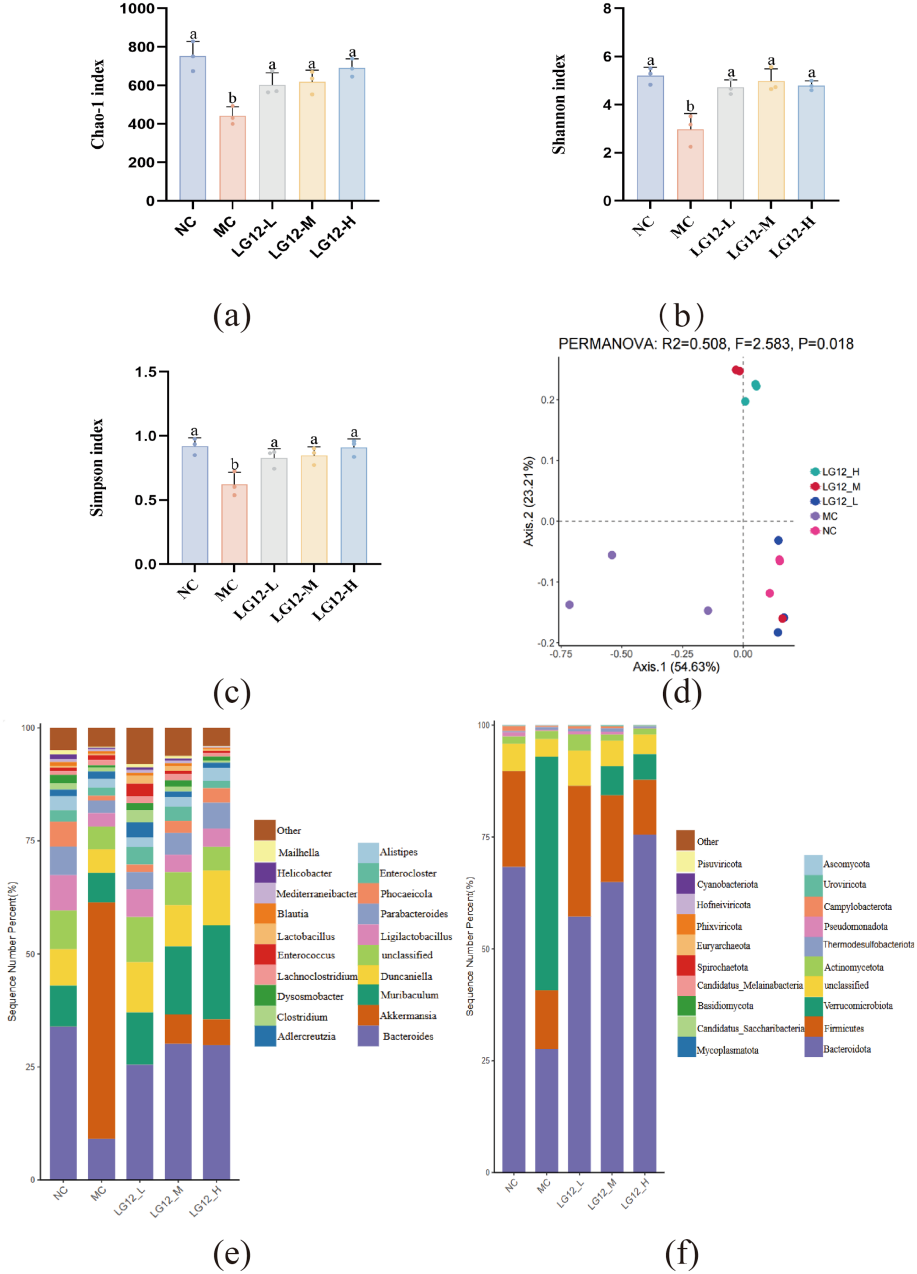
Intestinal flora composition in mice **(a)** Chao-1 index; **(b)** Shannon index; **(c)** Simpson index; **(d)** Beta diversity; **(e)** Relative abundance of gut microbiota in genus level; **(f)** Relative abundance of gut microbiota in phylum level. a–b: Different letters indicate (*p* < 0.05), and the same letters or no letters indicate no significant difference (*n* = 3).

In this paper, the *α*-diversity of the intestinal flora was reflected by the Chao1 index, the Shannon index and the Simpson index, with the Chao1 index indicating the abundance of the flora, and the Shannon and Simpson indices indicating the diversity of the community. As shown in Figures 5a–c, Compared with the NC group, the Chao1, Fisher and Shannon indices were significantly lower in the MC group (*p* < 0.05), which showed that the α-diversity of the intestinal flora was reduced in mice with loperamide-induced constipation. After *L. rhamnosus* Glory LG12 intervention, Chao1, Fisher and Shannon indices were significantly increased in all three dose groups (*p* < 0.05), which shows that *L. rhamnosus* Glory LG12 intervention restored the diversity and abundance of the intestinal flora of constipated mice to a certain extent.

Beta diversity analysis can visualise the differences in the composition of gut flora communities between different groups. According to the Bray-Curtis distance for principal coordinate analysis (PCoA). As shown in Figure 5d, the three sample points in the NC group were more aggregated, indicating that the community structure of mice in the NC group was more similar and the stability of the intestinal flora was higher. The three sample points of mice in the MC group were clearly separated from and distant from the NC group. In addition, the three sample points were distant from each other, suggesting that the bacterial flora within the MC group varied greatly among different individuals and that the intestinal flora within the constipated mice was disturbed. After the intervention of *L. rhamnosus* Glory LG12, the sample points of three groups, LG12-L, LG12-M and LG12-H had a significant tendency to converge to the NC mice, indicating that *L. rhamnosus* Glory LG12 improves the similarity of the community structure and the stability of the intestinal flora.

At the genus level, as shown in Figure 5e, the intestinal flora of the groups of mice was dominated by *Bacteroides*, *Akkermansia*, *Muribaculum*, *Duncaniella*, *Ligilactobacillus*, *Parabacteroides*, *Phocaeicola*, *Enterocloster* and *Alistipes* were predominant. The abundance of Bacteroides and Parabacteroides was significantly lower and the abundance of *Akkermansia* was significantly higher in the intestinal bacterial flora of the MC group compared to the NC group. *L. rhamnosus* Glory LG12 decreased the abundance of *Akkermansia* and increased the abundance of *Bacteroides*, *Parabacteroides* and *Ligilactobacillus* in the intestinal bacterial flora in LG12-L, LG12-M and LG12-H groups, compared to the MC group after the intervention of *L. rhamnosus* Glory LG12. Meanwhile, the abundance of *Muribaculum* and *Duncaniella* was significantly increased in the LG12-L, LG12-M and LG12-H groups after the intervention treatment with *L. rhamnosus* Glory LG12 compared to the MC group and was even higher than the related abundance in the NC group. These results suggest that the preventive effect of *L. rhamnosus* Glory LG12 on constipation may be related to its effect on the abundance of intestinal flora.

At the Phylum level, as shown in Figure 5f, the intestinal flora of mice in each group was mainly dominated by *Bacteroidota*, *Firmicutes*, *Verrucomicrobiota* and *Actinomycetota*. Compared with the NC group, the abundance of *Bacteroidota* and Firmicutes in the MC group was significantly lower, and the abundance of *Verrucomicrobiota* was significantly higher. *L. rhamnosus* Glory LG12 treatment increased the abundance of *Bacteroidota* and Firmicutes and decreased the abundance of *Verrucomicrobiota* in the defecation intestinal flora in the LG12-L, LG12-M and LG12-H groups compared to the MC group.

#### 3.6.2 Metabolic pathways in the macrogenome

**Figure 6:**
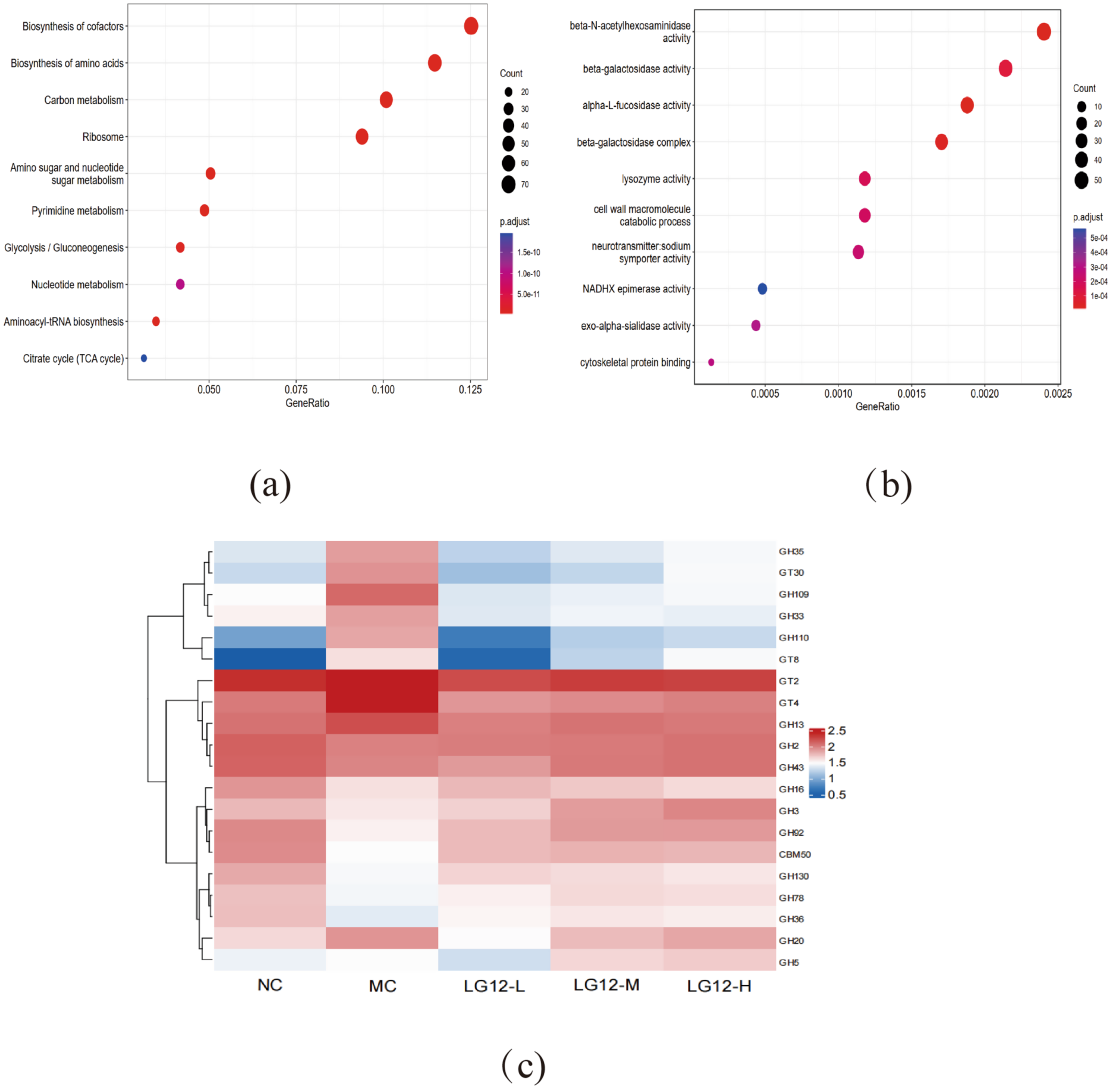
KEEG, GO and carbohydrate metabolic pathways of gut microorganisms. **(a)** MC and LG12-H groups: Differential gene KEGG enrichment analysis bubble plot; **(b)** MC and LG12-H groups: Differential gene GO enrichment analysis bubble plot. **(c)** Abundance analysis of carbohydrate-related metabolic pathways in the KEGG database. Colours range from red to blue, with red representing higher expression levels of certain genes. Bubble size is proportional to the number of genes enriched in the pathway (*n* = 3).

As shown in Figure 6a, KEGG enrichment analysis of differential genes in MC and LG12-H groups revealed that these differential genes were involved in various KEGG pathways, including Biosynthesis of cofactors, Biosynthesis of amino acids, Carbon metabolism, Ribosome, Amino sugar and nucleotide sugar metabolism, Pyrimidine metabolism and other pathways. As shown in Figure 6b, GO enrichment analysis of differential genes in MC and LG12-H groups revealed that differential genes were mainly involved in biological processes such as beta-N-acetylhexosaminidase activity, beta-galactosidase activity, alpha-L-fucosidase activity, beta-galactosidase activity, alpha-L-fucosidase activity, beta-galactosidase complex, NAD + kinase activity and other biological processes.

As shown in Figure 6c, glycosidases such as GH16, GH3, GH92, GH130, GH78 and GH36, etc. in the MC group had lower abundance in the CAZy database compared to the LG12-H group, suggesting that the related enzymes were less activated or less involved in the metabolic pathway.

### 3.7 Effect of *Lacticaseibacillus rhamnosus* Glory LG12 on SCFAS in mice

**Figure 7:**
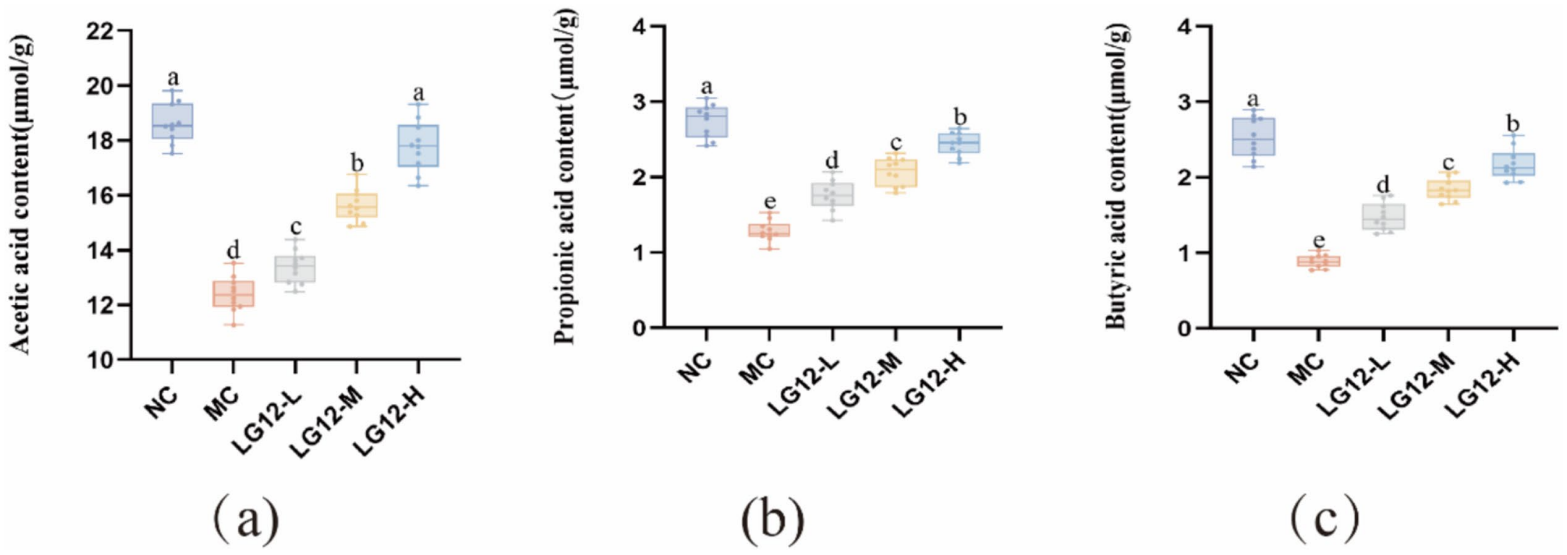
Effect of *L. rhamnosus Glory LG12* on short chain fatty acid content. **(a)** Acetic acid content; **(b)** Propionic acid content; **(c)** Butyric acid content. a–e: Different letters indicate (*p* < 0.05), and the same letters or no letters indicate no significant difference (*n* = 10).

As shown in Figure 7, the contents of acetic acid, propionic acid and butyric acid in the defecation of mice in the MC group were significantly reduced (*p* < 0.05) after gavage of loperamide-induced constipation. It is likely that loperamide-induced constipation caused disruption of the intestinal flora, affecting the structural composition of the intestinal flora and its metabolites. After the intervention of *L. rhamnosus* Glory LG12, the amounts of all three SCFA_S_ in the LG12-L, LG12-M, and LG12-H groups showed a significant increase (*p* < 0.05) compared with the MC group. The experimental results showed that the total content of SCFAs was positively correlated with the intervention dose. Among them, the butyric acid level of the LG12-H group was elevated 1.4-fold compared with the model group, which was significantly higher than that of the LG12-L group, further verifying that the restoration of the metabolic function of the mouse colony was dependent on the dose of *L. rhamnosus*. This suggests that *L. rhamnosus* Glory LG12 can increase the concentration of SCFA_S_ by increasing the concentrations of acetic acid, propionic acid, and butyric acid in the intestinal tract of mice, thereby treating constipation.

## 4 Discussion

Constipation is a very common gastrointestinal disease with a very high incidence rate, which seriously affects human health and is increasing year by year (Wang L. et al., 2020). When intestinal peristalsis is weakened, it leads to food and waste products staying in the intestine for too long, which can easily cause intestinal flora disorders. Dysregulation of intestinal microbial homeostasis leads to an imbalance in the composition and function of these intestinal microorganisms, triggering diseases such as constipation (Chai et al., 2021). Research has shown that probiotics can effectively relieve constipation by regulating intestinal flora and restoring intestinal ecological balance (Ghuloom et al., 2023). This experiment established a mouse constipation model by gavage of loperamide hydrochloride, and different dose groups were set up in the experiment. The aim is to explore the preventive effect and mechanism of action of *L. rhamnosus* Glory LG12 on constipated mice.

Loperamide hydrochloride inhibits peristalsis, prolongs the retention time of food residues in the intestine, and over-absorbs intestinal water, thus making the defecation dry and hard, prolonging the time of defecation elimination, and reducing the number of defecation pellets (Mori et al., 2013). The effect of *Lacticaseibacillus rhamnosus* on constipation can be evaluated by observing and measuring the defecation indexes (water content of defecation, time to first black stool defecation, small intestine transit rate, and number of grains in 5 h defecation) in mice. The small intestine transit rate can reflect the effect of probiotics on the intestinal motility of mice, the higher the propulsion rate, the stronger the peristalsis of the small intestine (He et al., 2022). The water content of defecation can reflect the degree of constipation and is also an important indicator of the effectiveness of medication in improving constipation. The time to first black stool defecation refers to the length of time from the beginning of the food from the stomach to the expulsion of the body in the form of defecation, which can reflect the peristaltic ability of the whole gastrointestinal tract. That is, the longer the first black stool time, the worse the peristaltic ability of mice The results of this experiment showed that the water content of defecation, the number of grains in 5 h defecation and the small intestine transit rate of mice in the MC group decreased significantly (*p* < 0.05), and the time to first black stool defecation increased significantly (*p* < 0.05), which indicated that the constipation model was successful in modelling. After gavage of different doses of *L. rhamnosus* Glory LG12, the constipated mice showed a significant increase in defecation water content, number of grains in 5 h defecation and small intestine transit rate, as well as a significant decrease in the time to the first black stool, and an improvement in defecation indexes, which indicated that *L. rhamnosus* Glory LG12 had the effect of preventing constipation. The 5 h defecation, defecation water content and small intestine transit rate were significantly higher in the LG12-L group than in the LG12-H group, and the time to the first black stool was significantly lower than that in the LG12-H group, which all indicated that the higher These results indicated that the higher dose of *L. rhamnosus* Glory LG12 had a better effect on preventing constipation in a dose-dependent manner. Yang et al. (2020) results showed that *Lactobacillus paracasei* LC2 (*L. paracasei* LC2) significantly reduced the time to first black stool in constipated mice. Wang et al. (2017) showed that the *Bifidobacterium adolescentis* CCFM 667 and 669 (*B. adolescentis* CCFM 667, *B. adolescentis* CCFM 669) dosage groups showed a significant increase in defecation weight, number of defecation grains, water content of defecation, and small intestine transit rate when compared to the control group (Loperamide hydrochloride group). Meanwhile, the high dose group of *B. adolescentis* CCFM 667 and 669 groups showed significant improvement in constipation compared to the low dose group. This is consistent with the results obtained in this experiment.

By HE staining, the structure and morphology of colonic tissues can be visualised, which is helpful to directly determine whether *Lacticaseibacillus rhamnosus* has a reparative effect on pathological intestinal damage. In the present study, inflammatory infiltration of colonic tissues was improved and intestinal glandular arrangement was largely restored to normal after *L. rhamnosus* Glory LG12 intervention. Li et al. (2024) gavage of *Lacticaseibacillus paracasei* NCU-04 (*L. paracasei* NCU-04) to constipated mice showed that L. paracasei NCU-04 attenuated pathological changes in the colon of constipated mice. Li et al. (2021) investigated the effects of probiotic yoghurt containing konjac mannan oligosaccharide (KMOS) and *Bifidobacterium animalis* BB12 (*B. animalis* BB12) on constipated mice. The HE results showed that the YBK group could effectively ameliorate the damage and inflammatory infiltration of the villous structure in constipated mice, and restore the intestinal tissues of mice to normal.

Constipation can lead to changes in neurotransmitters that inhibit the movement of intestinal muscles, thus weakening bowel movements (Wang et al., 2019). Common serum neurotransmitters include MTL, GAS, SP, ACH, VIP, SS, PYY, 5-HT, etc. Among them, MTL, GAS, SP, ACH belong to the excitatory neurotransmitters, and excitatory transmitters can promote gastrointestinal motility (Li et al., 2023); VIP, SS, PYY, and 5-HT are inhibitory neurotransmitters, and inhibitory transmitters inhibit the contraction of small intestinal smooth muscle, which in turn leads to constipation (Ballantyne, 2006). 5-HT is a key factor in the regulation of neurotransmitters, stimulating CGRP secretion and thus regulating the levels of gastrointestinal hormones and neurotransmitters (Mawe et al., 2022). MTL is a hormone in the digestive tract that plays a role in promoting gastrointestinal motility and the transport of water and electrolytes in the gastrointestinal tract. ET is closely related to vascular constriction (Guo et al., 2023). Inhibitory brain gut peptide SS regulates the contractile function of gastrointestinal smooth muscle. SS secretion promotes the secretion of vasoactive peptide VIP and neurotransmitter SP, leading to smooth muscle relaxation and subsequently affecting bowel movements. SP is a neurotransmitter distributed throughout nerve fibers that can stimulate sensation and movement in the intestine (Zhou et al., 2022). GAS can be increased through mechanical or chemical stimulation, thereby increasing sphincter tension and promoting gastrointestinal peristalsis (Yi et al., 2022). Li et al. (2014) showed that the levels of MTL, Gas, and ACHe in constipated mice were lower than those in normal control mice, and the levels of MTL, Gas, and ACHe were greatly elevated after PLCSB administration. Li et al. (2021) showed that synthetic yoghurt supplemented with 2.0% KMOS and BB12 reduced serum levels of inhibitory neurotransmitter VIP and excitatory neurotransmitter (Ach, MTL and SP) content. As can be seen from the experimental results, the levels of excitatory transmitters in constipated mice were significantly decreased (*p* < 0.05) and the levels of inhibitory transmitters were significantly increased (*p* < 0.05) (Li et al., 2024).

IL-1β, IL-6, IL-8, IFN-*γ*, TNF-*α*, IL-10 are major regulators of immune response and inflammation (Mühl and Pfeilschifter, 2003). As the intervention dose increased, the levels of pro-inflammatory factors showed a significant decreasing trend. Compared with the MC group, the LG12-L group only slightly decreased IL-6 (by 11.57%,) and IFN-γ (by 14.48%), whereas the LG12-H group significantly suppressed the inflammatory factors (IL-1β decreased by 35.4%, IL-6 decreased by 34.7%, and IFN-γ decreased by 31.25%). The effect of the LG12-M group was in between (IL-1β decreased by 25.72%, IL-6 decreased by 25.7%, and IFN-γ decreased by 22.78%), suggesting that LG12-L group had a significant decrease in inflammatory factors. The effect of LG12-M group was in between (IL-1β decreased by 25.72%, IL-6 decreased by 25.7%, and IFN-γ decreased by 22.78%), suggesting a dose-dependent effect of *L. rhamnosus* Glory LG12 on the prevention of constipation. Zhang et al. (2023) showed that all probiotic-treated groups significantly down-regulated serum IL-1, IL-6, and IL-8 levels and up-regulated IL-10 levels in constipated mice when compared with the model group. In conclusion, is consistent with the results of this experiment.

In the experiment, the *α*-diversity of the constipated mice group was significantly lower (*p* < 0.05), whereas the α-diversity of the three groups treated with *L. rhamnosus* Glory LG12 was significantly higher (*p* < 0.05), which indicated that *L. rhamnosus* Glory LG12 treatment restored the diversity and abundance of the intestinal flora of the constipated mice to some extent. However, there was no significant difference in alpha diversity among the three groups of *L. rhamnosus* Glory LG12 gavage. This may be because, the shorter intervention time of this experiment may not be sufficient to trigger a significant change in the α-diversity of the intestinal flora. Wei Y. et al. (2023) conducted an experimental study on constipation in which mice, except for the normal control group, were treated with 5 mg/kg loperamide for 1 week, and then treated with hawthorn, probiotics and hawthorn postbiotics-prebiotics for another week. The results showed that there was no statistically significant alpha diversity between the normal control group, the model group and the hawthorn postbiotic-probiotic group. In addition, the constipation model used in this experiment may preferentially affect specific functional flora rather than the overall diversity, which is in line with the observed variation in *β*-diversity but stability in α-diversity in the study by Liang et al. (2023a).

PcoA analysis based on the Bray-Curtis distance matrix showed that the sample points of mice in the MC group were significantly separated and distant from those of the NC group, suggesting that the intestinal flora in constipated mice was disturbed. The sample points of LG12-L, LG12-M and LG12-H groups significantly converged to those of the NC group, suggesting that *L. rhamnosus* Glory LG12 improved the similarity of the community structure and the stability of the intestinal flora. The proximity in spatial location of one sample point between the NC and LG12-M groups suggests a high degree of similarity in multidimensional characteristics between the 2 groups. On the one hand, it may be due to sample differences, and on the other hand, the proximity between the LG12-M group and the NC group may indicate that the abundance of certain key bacterial genera has approached normal, but the overall community has not yet reached a stable state as in the high-dose group. Li et al. (2024) also obtained similar results.

Studies have shown that the formation of constipation is associated with disturbances in the intestinal flora, while the restoration of intestinal flora homeostasis can help to improve constipation (Yarullina et al., 2020). Therefore, it is necessary to explore the changes of intestinal flora in the colon of constipated mice. Elevated abundance of *Verrucomicrobia* in the defecation of patients with slow-transmission constipation (Ge et al., 2017), consistent with the results obtained in this experiment. Wang G. et al. (2020) showed increased abundance of *Akkermansia* in mice defecation, which is consistent with the results of this experiment. *Akkermansia* can utilise mucin in the human gut as the sole source of carbon and nitrogen, whereas a reduction in mucin affects the barrier function of the gut, as well as drying out the intestinal contents (Derrien et al., 2004). The results of this experiment showed that after treatment with *L. rhamnosus* Glory LG12, there was a significant increase in the abundance of *Bacteroidota* and *Firmicutes* in constipated mice at the phylum level; and at the genus level, there was a significant increase in the abundance of *Bacteroides*, *Muribaculum*, *Ligilactobacillus and Parabacteroides* in constipated mice. The discussion has already addressed the relationship between upregulated microbiota and constipation. *L. rhamnosus* LRJ-1 treatment significantly enhanced the abundance of *Bacteroides*. in constipated mice, but decreased the abundance of *Akkermansia*. Indicating that *L. rhamnosus* strain LRJ-1 relieves constipation by enriching anabolic bacilli (Xia et al., 2024). *Bacteroides* are major members of the gut microbiota in thermostats producing acetate, propionate and succinate, and have been reported to be reduced in constipated mice (Huang et al., 2022). *Bacteroides* can stimulate intestinal motility by increasing intestinal *γ*-aminobutyric acid, vesicle-associated protein-33 and γ-actin expression (Zhang X. et al., 2021). *Ligilactobacillus* secretes antimicrobial molecules such as organic acids, ethanol and lutrophilic lipoproteins. *Ligilactobacillus* increases gut levels of butyrate, which plays a wide range of roles in local and whole organism signalling networks by binding to G protein-coupled receptors (GPCRs) (Guo et al., 2025). *Parabacteroides* are the producer of SCFAs (Liang et al., 2023a). SCFAs can regulate the water content in feces by acting as an anionic osmoregulator in the intestine, and regulate intestinal wall peristalsis by modulating the release of gastrointestinal hormones (Zhang et al., 2025).

Studies have shown that firmicutes are positively correlated with the pathways of H_2_ and CH_4_ production, which can stimulate the enteric nervous system and thus promote peristalsis (Mancabelli et al., 2017). Bacteroides modulates tryptophan synthesis in germ-free mice, which is an important precursor for the synthesis of 5-HT, thus stimulating gastrointestinal peristalsis and relieving constipation (Bhattarai et al., 2018). In this study, it was hypothesised that restoring the abundance of *Firmicutes* and *Bacteroides* may be an important pathway for the preventive of constipation by *L. rhamnosus* Glory LG12.

Intestinal flora and metabolic processes contribute to the maintenance of a stable intestinal microenvironment and normal physiological functions. In this experiment, the biochemical metabolic pathways of constipation were identified by enriching differential genes through KEGG pathway and GO entries. The results showed that the treatment of constipation by *L. rhamnosus* Glory LG12 was associated with the regulation of Biosynthesis of cofactors, Biosynthesis of amino acids, Carbon metabolism, Ribosome, Amino sugar and nucleotide sugar metabolism, Pyrimidine metabolism, beta-N-acetylhexosaminidase activity, beta-galactosidase activity, alpha-L-fucosidase activity, beta- galactosidase activity, alpha-L-fucosidase activity, beta-galactosidase complex, NAD + kinase activity and other biological processes are closely related to metabolic pathways.

Pyrimidine metabolism is in a state of balance in the body, and when metabolic disorders occur, they can lead to a series of diseases, such as neurological disorders, circulatory disorders, and immune system-related diseases (Sun et al., 2021). It has been shown that pyrimidine metabolism is disrupted in constipated mice and that modulation of pyrimidine metabolism reduces intestinal inflammation and barrier damage (Wang et al., 2024). In this experiment, gavage of a high dose of *L. rhamnosus* Glory LG12 effectively restored pyrimidine metabolic dysregulation and regulated intestinal damage, thereby alleviating constipation symptoms. Zhang Q. et al. (2021) hypothesised that konjac glucomannan could alleviate constipation by regulating metabolic pathways such as the biosynthesis of amino acids (phenylalanine, tyrosine and tryptophan). Beta-galactosidase is an important enzyme in the intestinal microbiota. Lower production and activity of beta-galactosidase in patients with constipation may interfere with the digestion and absorption of lactose and other fermentable carbohydrates, which may lead to a build-up of undigested material in the intestinal tract, increasing the burden on the intestinal tract and interfering with normal peristalsis, which may in turn exacerbate the symptoms of constipation. Bouhnik et al. (2004) showed an increase in *β*-galactosidase activity in patients with chronic idiopathic constipation after 28 d of lactulose treatment, which is consistent with the results of this experiment.

In addition to this, we also investigated the expression of various groups of glycolytic enzymes in the CAZy database, with mouse gut microbes expressing mainly glycoside hydrolases (GHs) and glycosyltransferases (GTs). Glycoside hydrolases play an important role in the study of constipation, as they influence the composition and function of the intestinal flora, which in turn regulates intestinal health and defecation. More genes in the glycosyl hydrolase family could mean a greater ability to utilise complex carbohydrates and produce SCFAs (Wei X. et al., 2023). Microorganisms in the gut (e.g., bacteria, fungi, etc.) can break down complex carbohydrates such as dietary fibre, cellulose, hemicellulose, etc., into simple sugars by means of glycoside hydrolases, which are further catabolised and metabolised into pyruvic acid, which is then passed through a number of biochemical pathways, ultimately resulting in the production of SCFAs (Krautkramer et al., 2021).

SCFAs also known as volatile fatty acids, mainly include acetic acid, propionic acid and butyric acid. The production of SCFA_S_ leads to a decrease in PH, which reduces the growth of pathogenic bacteria and is beneficial to intestinal health (Yin et al., 2018). In addition, SCFAs can increase the osmotic pressure in the intestines, promote the absorption of water in the intestines, and stimulate the contraction of intestinal smooth muscle, which in turn promotes intestinal peristalsis and relieves constipation (Calderon et al., 2020). Therefore, the effectiveness of *L. rhamnosus* Glory LG12 in relieving constipation can be determined by measuring the SCFAs content in defecation. Some studies have shown that acetic acid has the ability to regulate intestinal motility and relieve constipation (Niwa et al., 2002). Propionic acid has some anti-inflammatory properties that may help reduce intestinal inflammation and promote the absorption of water and electrolytes. Dias et al. (2021) showed that butyrate provides energy to the intestinal epithelium, which can enhance the barrier function of the intestinal epithelium, reduce intestinal inflammation, and help to improve symptoms associated with constipation. In Zheng et al. (2023a) study, subjects with functional constipation who took a probiotic product (containing *Lactobacillus acidophilus* NCFM, *Bifidobacterium lactis* HN019, *Bifidobacterium lactis* Bl-04, *Bifidobacterium lactis* B420, *Lactobacillus plantarum* Lp-115, and *Lactobacillus paracasei* Lpc-37, and prebiotics) for 4 weeks showed an increase in the levels of all three major SCFA_S_ (acetic, propionic, and butyric) and relief of gastrointestinal symptoms. Were increased, the subjects had an increased number of bowel movements per week, and gastrointestinal symptoms were relieved. The results of this experiment showed that all three groups of *L. rhamnosus* Glory LG12 by gavage showed an increasing trend in the content of acetic acid, propionic acid, and butyric acid compared with the model group (*p* < 0.05). Wang et al. (2019) study showed that the concentration of SCFAs is important in relieving constipation. Among them, the combination of adherent bifidobacteria produces more propionic acid and butyric acid, which is more favourable to improve the symptoms of constipation. Zheng et al. (2020) showed that SCFAS were closely associated with *Lacticaseibacillus rhamnosus* repair of intestinal tight junctions and reduction of pro-inflammatory factor levels. In this experiment, after gavage of *L. rhamnosus* Glory LG12, the levels of pro-inflammatory factors decreased significantly in all three dose groups. Therefore, it is possible that *L. rhamnosus* Glory LG12 inhibits the level of pro-inflammatory factors by increasing the level of SCFA_S_, which in turn treats constipation.

In summary, *L. rhamnosus* Glory LG12 can effectively prevent constipation-related symptoms in mice. However, further studies are needed to elucidate the mechanism of *L. rhamnosus* Glory LG12 in preventing constipation symptoms by combining metabolomics and immunological techniques. Existing studies mostly explain the mechanism of probiotic treatment of constipation in mice from the perspective of intestinal interactions, and more perspectives, such as the gut-brain axis, may be needed in the future to elucidate the mechanism of probiotics in the treatment of Constipation.

## 5 Conclusion

The results showed that *L. rhamnosus* Glory LG12 restored constipation in mice by promoting intestinal peristalsis, repairing intestinal damage, down-regulating the levels of anti-inflammatory factors, and up-regulating the levels of pro-inflammatory factors. After intervention with *L. rhamnosus* Glory LG12, On the one hand, it upregulated the abundance of *Bacteroidota*, *Firmicutes*, *Bacteroides*, *Ligilactobacillus*, and *Parabacterioids* in the gut microbiota; On the other hand, regulating different metabolic pathways such as amino acid biosynthesis and pyrimidine metabolism promotes the balance of gut microbiota. At the same time, it also promotes the expression of various glycoside hydrolases, increases the generation of SCFAs, lowers the pH value of defecation, inhibits the level of pro-inflammatory factors, stimulates gastrointestinal peristalsis in mice, and thereby preventing constipation.

In conclusion, *L. rhamnosus* Glory LG12 can effectively regulate constipation traits in mice. Meanwhile, with the increase of the dose of *Lacticaseibacillus rhamnosus*, the relief effect on constipation was more significant. Overall, *L. rhamnosus* Glory LG12 demonstrated its potential as a probiotic preparation for the prevention of constipation. Among them, SCFAS, intestinal flora, and metabolic pathways such as pyrimidine metabolism, and amino acid biosynthesis play important roles in regulating constipation. In the future, more attention could be paid to the role of short-chain fatty acids, gut flora and metabolic pathways in the treatment of constipation.

## Data Availability

The original contributions presented in the study are publicly available. This data can be found here: [https://dataview.ncbi.nlm.nih.gov/PRJNA1274847].
