# Correction: *Lacticaseibacillus rhamnosus* Glory LG12 preventives loperamide-induced constipation in mice by modulating intestinal flora and metabolic pathways

**DOI:** 10.3389/fmicb.2025.1676952

**Published:** 2025-08-14

**Authors:** Weiwei Ma, Lian Lian, Lidong Guo, Yanan Wu, Lili Huang

**Affiliations:** College of Pharmacy, Heilongjiang University of Chinese Medicine, Harbin, Heilongjiang, China

**Keywords:** *Lacticaseibacillus rhamnosus* Glory LG12, constipation, intestinal flora, short-chain fatty acids, metabolic pathway

In the published article, there was an error in the **article title**.

The title of this article was erroneously given as:

“*Lactobacillus rhamnosus* Glory LG12 preventives loperamide-induced constipation in mice by modulating intestinal flora and metabolic pathways”,

The correct title should be:

“*Lacticaseibacillus rhamnosus* Glory LG12 preventives loperamide-induced constipation in mice by modulating intestinal flora and metabolic pathways”.

There was a mistake in the caption of **Figure 1, page 5** as published. The position of “a–e: Different letters indicate (p < 0.05), and the same letters or no letters indicate no significant difference (n = 10)” is incorrect.

The corrected caption of **Figure 1** appears below:

“Effect of *L. rhamnosus* Glory LG12 on defecation index in mice. **(a)** Time to the first black stool defecation; **(b)** Number of grains in 5 h defecation; **(c)** Water content of defecation; **(d)** Small intestine transit rate. a–e: Different letters indicate (*p* < 0.05), and the same letters or no letters indicate no significant difference (*n* = 10).”

There was a mistake in the caption of **Figure 2**, **page 7** as published. The legend for Figure 2 is incomplete, as it does not include the statement “a–d: Different letters indicate (p < 0.05), and the same letters or no letters indicate no significant difference (n = 3).

The corrected caption of **Figure 2** appears below:

“Effect of *L. rhamnosus* Glory LG12 on pathological changes in mice colon tissues (original magnification × 200) and colonic pathology score. **(a)** Pathological changes in colon tissues, **(b)** Colonic pathology score. a–d: Different letters indicate (*p* < 0.05), and the same letters or no letters indicate no significant difference (*n* = 3).”

“*Lactobacillus rhamnosus* Glory LG12” used in the article needs to be updated to the latest strain name “*Lacticaseibacillus rhamnosus* Glory LG12”.

A correction has been made to the **Citation, page 1**.

The Citation previously stated:

“Ma W, Lian L, Guo L, Wu Y and Huang L (2025) Lactobacillus rhamnosus Glory LG12 preventives loperamide-induced constipation in mice by modulating intestinal flora and metabolic pathways. *Front. Microbiol*. 16:1577799. doi: 10.3389/fmicb.2025.1577799

The corrected Citation appears below:

“Ma, W., Lian, L., Guo, L., Wu, Y., and Huang, L. (2025) *Lacticaseibacillus rhamnosus* Glory LG12 preventives loperamide-induced constipation in mice by modulating intestinal flora and metabolic pathways. *Front. Microbiol*. 16:1577799. doi: 10.3389/fmicb.2025.1577799”

A correction has been made to **1 Introduction, page 2**.

This sentence previously stated:

“*Lactobacillus rhamnosus* has been shown to regulate intestinal flora disorders and maintain intestinal microecology in mice (Mei et al., 2024). Han et al. (2024) administered *Lactobacillus rhamnosus* JYLR-127 (*L. rhamnosus* JYLR-127) to patients with constipation after bone fracture, and the results showed that the administration of *Lactobacillus rhamnosus* was beneficial to the reconstruction of the destroyed intestinal flora and the modification of intestinal microecology. He et al. (2022) detected a significant increase in the abundance of *Lactobacillus rhamnosus* in the intestinal tract of mice after stopping gavage of *Lactobacillus rhamnosus* LR22 (*L. rhamnosus* LR22) for 2 weeks, suggesting that *Lactobacillus rhamnosus* colonised the intestinal tract of mice. The results showed that high-dose *Lactobacillus rhamnosus* had better colonization effects than low-dose *Lactobacillus rhamnosus*, thereby better regulating intestinal microbiota and relieving constipation.”

The corrected sentence appears below:

“*Lacticaseibacillus rhamnosus* has been shown to regulate intestinal flora disorders and maintain intestinal microecology in mice (Mei et al., 2024). Han et al. (2024) administered *Lactobacillus rhamnosus* JYLR-127 (*L. rhamnosus* JYLR-127) to patients with constipation after bone fracture, and the results showed that the administration of *Lacticaseibacillus rhamnosus* was beneficial to the reconstruction of the destroyed intestinal flora and the modification of intestinal microecology. He et al. (2022) detected a significant increase in the abundance of *Lacticaseibacillus rhamnosus* in the intestinal tract of mice after stopping gavage of *Lactobacillus rhamnosus* LR22 (*L. rhamnosus* LR22) for 2 weeks, suggesting that *Lacticaseibacillus rhamnosus* colonised the intestinal tract of mice. The results showed that high-dose *Lacticaseibacillus rhamnosus* had better colonization effects than low-dose *Lacticaseibacillus rhamnosus*, thereby better regulating intestinal microbiota and relieving constipation.”

A correction has been made to **1 Introduction**, **page 3**.

This sentence previously stated:

“Available studies have shown that *Lactobacillus rhamnosus* Glory *LG12* (*L. rhamnosus* Glory LG12) can significantly increase the concentrations of lactic acid, acetic acid and butyric acid in the intestinal tract of mice (Ma et al., 2024).”

The corrected sentence appears below:

“Available studies have shown that *Lacticaseibacillus rhamnosus* Glory *LG12* (*L. rhamnosus* Glory LG12) can significantly increase the concentrations of lactic acid, acetic acid and butyric acid in the intestinal tract of mice (Ma et al., 2024).”

A correction has been made to **2 Materials and methods**, *2.2 Lactobacillus rhamnosus Glory LG12 suspension preparation*, **page 2**.

This heading previously stated:

“2.2 Lactobacillus rhamnosus Glory LG12 suspension preparation”

The corrected heading appears below:

“2.2 *Lacticaseibacillus rhamnosus* Glory LG12 suspension preparation”

A correction has been made to **3 Results**, *3.1 Effect of Lactobacillus rhamnosus Glory*

*LG12 on body weight in mice*, **page 5**.

This heading previously stated:

“3.1 Effect of Lactobacillus rhamnosus Glory LG12 on body weight in mice”

The corrected sentence appears below:

“3.1 Effect of *Lacticaseibacillus* rhamnosus Glory LG12 on body weight in mice”

A correction has been made to **3 Results**, *3.2 Effect of Lactobacillus rhamnosus Glory*

*LG12 on defecation indices in mice*, **page 5**.

This heading previously stated:

“3.2 Effect of Lactobacillus rhamnosus Glory LG12 on defecation indices in mice”

The corrected heading appears below:

“3.2 Effect of *Lacticaseibacillus rhamnosus* Glory LG12 on defecation indices in mice”

A correction has been made to **3 Results**, *3.3 Effect of Lactobacillus rhamnosus Glory*

*LG12 on pathological changes in mice colon tissue*, **page 5**.

This heading previously stated:

“3.3 Effect of Lactobacillus rhamnosus Glory LG12 on pathological changes in mice

colon tissue”

The corrected heading appears below:

“3.3 Effect of *Lacticaseibacillus rhamnosus* Glory LG12 on pathological changes in mice colon tissue”

A correction has been made to **3 Results**, *3.4 Effect of Lactobacillus rhamnosus Glory LG12 on serum neurotransmitters in mice***, page 6**.

This heading previously stated:

“3.4 Effect of Lactobacillus rhamnosus Glory LG12 on serum neurotransmitters in mice”

The corrected heading appears below:

“3.4 Effect of *Lacticaseibacillus rhamnosus* Glory LG12 on serum neurotransmitters in mice”

A correction has been made to **3 Results**, *3.5 Effect of Lactobacillus rhamnosus Glory*

*LG12 on inflammatory factors in mice*, **page 7**.

This heading previously stated:

“3.5 Effect of Lactobacillus rhamnosus Glory LG12 on inflammatory factors in mice”

The corrected sentence appears below:

“3.5 Effect of *Lacticaseibacillus rhamnosus* Glory LG12 on inflammatory factors in mice”

A correction has been made to **3 Results**, *3.6 Effect of Lactobacillus rhamnosus Glory*

*LG12 on intestinal flora in mice*, **page 8**.

This heading previously stated:

“3.6 Effect of Lactobacillus rhamnosus Glory LG12 on intestinal flora in mice”

The corrected heading appears below:

“3.6 Effect of *Lacticaseibacillus rhamnosus* Glory LG12 on intestinal flora in mice”

A correction has been made to **3 Results**, *3.7 Effect of Lactobacillus rhamnosus Glory*

*LG12 on SCFAS in mice*, **page 9**.

This heading previously stated:

“3.7 Effect of Lactobacillus rhamnosus Glory LG12 on SCFAS in mice”

The corrected heading appears below:

“3.7 Effect of *Lacticaseibacillus rhamnosus* Glory LG12 on SCFAS in mice”

A correction has been made to **4 Discussion**, **page 12**.

This sentence previously stated:

“The effect of *Lactobacillus rhamnosus* on constipation can be evaluated by observing and measuring the defecation indexes (water content of defecation, time to first black stool defecation, small intestine transit rate, and number of grains in 5 h defecation) in mice.”

The corrected sentence appears below:

“The effect of *Lacticaseibacillus rhamnosus* on constipation can be evaluated by observing and measuring the defecation indexes (water content of defecation, time to first black stool defecation, small intestine transit rate, and number of grains in 5 h defecation) in mice.”

A correction has been made to **4 Discussion, page 12**.

This sentence previously stated:

“By HE staining, the structure and morphology of colonic tissues can be visualised, which is helpful to directly determine whether *Lactobacillus rhamnosus* has a reparative effect on pathological intestinal damage.”

The corrected sentence appears below:

“By HE staining, the structure and morphology of colonic tissues can be visualised, which is helpful to directly determine whether *Lacticaseibacillus rhamnosus* has a reparative effect on pathological intestinal damage.”

A correction has been made to **4 Discussion, page 12**.

This sentence previously stated:

“Li et al. (2024) gavage of *Lactobacillus paracasei* NCU-04 (*L. paracasei* NCU-04) to constipated mice showed that *L. paracasei* NCU-04 attenuated pathological changes in the colon of constipated mice.”

The corrected sentence appears below:

“Li et al. (2024) gavage of *Lacticaseibacillus paracasei* NCU-04 (*L. paracasei* NCU-04) to constipated mice showed that *L. paracasei* NCU-04 attenuated pathological changes in the colon of constipated mice.”

A correction has been made to **4 Discussion**, **page 14**.

This sentence previously stated:

“Zheng et al. (2020) showed that SCFAS were closely associated with *Lactobacillus rhamnosus* repair of intestinal tight junctions and reduction of pro-inflammatory factor levels.”

The corrected sentence appears below:

“Zheng et al. (2020) showed that SCFAS were closely associated with *Lacticaseibacillus rhamnosus* repair of intestinal tight junctions and reduction of pro-inflammatory factor levels.”

A correction has been made to **5 Conclusion, page 15**.

This sentence previously stated:

“Meanwhile, with the increase of the dose of *Lactobacillus rhamnosus*, the relief effect on constipation was more significant.”

The corrected sentence appears below:

“Meanwhile, with the increase of the dose of *Lacticaseibacillus rhamnosus*, the relief effect on constipation was more significant.”

The original article has been updated.

