# Supplementary material for: Lacticaseibacillus rhamnosus Glory LG12 preventives loperamide-induced constipation in mice by modulating intestinal flora and metabolic pathways
Source: Front Microbiol. 2025 Jul 11;16:1577799. doi: 10.3389/fmicb.2025.1577799 (PMC12289570; doi:10.3389/fmicb.2025.1577799)
Supplement: Supplementary file 1 [file Table_1.docx]

***Lactobacillus rhamnosus Glory LG12 Genome Analysis Report***

**1、Basic information about the genome**

*Lactobacillus rhamnosus* Glory LG12 (*Lactobacillus rhamnosus* Glory LG12=YMYSL9-58) was assembled to obtain the whole genome, and the basic information of its genome was counted, and it was found that the genome size, the number of CDSs, and the number of RNAs in the *Lactobacillus rhamnosus* Glory LG12 genome were more consistent with the Probio-M9 and GG were more consistent. The genome size of *Lactobacillus rhamnosus* Glory LG12 was 3.11 Mb and there were 2946 CDS.

**Table 1 Basic information about the genome**

| Strain | Size (bp) | GC (%) | RNAs | CDS |
| --- | --- | --- | --- | --- |
| HN001 | 2,914,408 | 46.7 | 53 | 2886 |
| Probio-M9 | 2,987,632 | 46.8 | 75 | 2934 |
| GG | 3,010,116 | 46.7 | 72 | 2983 |
| Lactobacillus rhamnosus Glory LG12 | 3,114,793 | 46.7 | 77 | 2946 |

**2、Phylogenetic relationships and SNP analysis**

The phylogenetic tree was constructed with the obtained 2126 core genes using the neighbour-joining method, and *Lactobacillus rhamnosus* DSM20021^T^ was divided into 2 branches with other strains, in which HN001 was clustered in the same branch with GG, and LR-109, Probio-M9 were genetically closer to DSM20021^T^ and clustered in the same branch.

Mauve covariance analysis was performed using Probio-M9 as the reference strain. *Lactobacillus rhamnosus* Glory LG12 was highly similar to BB-12, V9 and Probio-M8, with only localised fragment insertions. In addition, the number of SNPs of *Lactobacillus rhamnosus* Glory LG12 was 21 with Probio-M8, 43 with V9 and 34 with BB-12, and 13 unique genes were found in the genome of *Lactobacillus rhamnosus* Glory LG12 were (epsL, rfaB, rfbX, yiaC, dld, menB, menE, sauU, nfrA, xylA, nanK), which were mainly associated with immunomodulation and polysaccharide synthesis. The above results indicate that the genomes of *Lactobacillus rhamnosus* Glory LG12 differed significantly from those of Probio-M9, GG, and HN001.

**
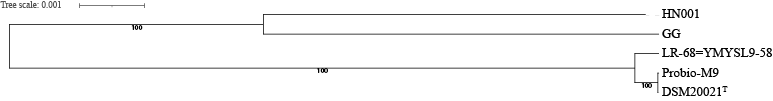
**

**Fig. 2 Phylogenetic tree
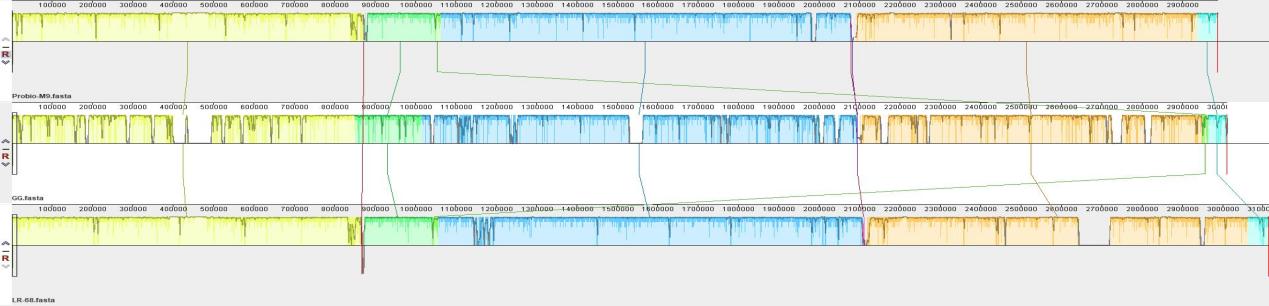
**

**Fig. 3 Mauve covariance analysis**

**Table 2 SNP analysis**

|  | Probio-M9 | GG |
| --- | --- | --- |
| Lactobacillus rhamnosus Glory LG12 | 5923 | 64,805 |

**3、Tolerance gene analysis**

The key to maintaining the viability of Lactobacillus is to increase the stress tolerance of Lactobacillus to withstand a range of environmental stresses such as extremes of temperature, osmolality, pH, and oxygen. Tolerance-related functional genes were annotated for *Lactobacillus rhamnosus* Glory LG12, Probio-M9, GG, and HN001, and *Lactobacillus rhamnosus* Glory LG12, Probio-M9, GG, and HN001 contained consistent tolerance-related genes. Eleven acid tolerance-related genes (*lys*C, *dap*A, *arg*C, *arg*H, *met*E, *arg*R, *arg*J, *arg*S, *aax*C, *mur*E, *lys*S), nine heat tolerance-related genes (*dna*K, *hrc*A, *dna*J, *grp*E, *dna*A, *dna*G, *dna*B, *dna*E, *dna*N), and one gene (*dna*B, *dna*E, *dna*N) were annotated. tolerance-related genes (*oxy*R); two cold tolerance-related genes (*csp*A, *clp*C); and three bile salt tolerance-related genes (*cbh*, *gly*QS, *pur*D, *gcv*H).

**Table 2 Results of tolerance-related genes**

| Acid Resistance Related Genes | | | |
| --- | --- | --- | --- |
| *arg*S | Arginine--tRNA ligase | *arg*R | Arginine repressor |
| *dap*A | 4-hydroxy-tetrahydrodipicolinate synthase | *arg*H | Argininosuccinate lyase |
| *met*E | 5-methyltetrahydropteroyltriglutamate--homocysteine methyltransferase | *mur*E | UDP-N-acetylmuramoyl-L-alanyl-D-glutamate--L-lysine ligase |
| Heat resistance-related genes | | | |
| *dna*K | Chaperone protein DnaK | *grp*E | Protein GrpE |
| *hrc*A | Heat-inducible transcription repressor HrcA | *dna*A | Chromosomal replication initiator protein DnaA |
| *dna*J | Chaperone protein DnaJ | *dna*G | DNA primase |
| *dna*N | Beta sliding clamp | *dna*B | Replicative DNA helicase |
| *dna*E | DNA polymerase III subunit alpha |  |  |
| Oxygen tolerance-related genes | | | |
| *oxy*R | | Hydrogen peroxide-inducible genes activator | |
| Bile salt tolerance-related genes | | | |
| *gcv*H | Glycine cleavage system H protein | *pur*D | Phosphoribosylamine--glycine ligase |
| Cold tolerance-related genes | | | |
| *clp*C | | putative ATP-dependent Clp protease ATP-binding subunit | |

**4、Probiotic-related gene** **analysis**

*Lactobacillus rhamnosus* Glory LG12, Probio-M9, GG and HN001 genomes were annotated for probiotic-related genes, and functional genes related to riboflavin, lactic acid, production of extracellular polysaccharides, synthesis of bioactive peptides, and acid and bile salts tolerance were obtained, and *Lactobacillus rhamnosus* Glory LG12, Probio-M9, GG and HN001 contained the same number of copies of functional genes. genes with the same copy number.

The above strains were used for probiotic prediction using the iProbiotics platform (there are more than 2282 probiotic strains enriched genes in the iProbiotics platform). Analysis using Model1:Probiotic Predictor of the iProbiotics platform revealed that the probability of *Lactobacillus rhamnosus* Glory LG12 being a probiotic was 99.89% in agreement with the prediction results of Probio-M9 and GG.

**Table 3 Probiotic-related genes**

| Functional | Gene | Annotation | Lactobacillus rhamnosus Glory LG12 | Probio-M9 | GG | HN001 |
| --- | --- | --- | --- | --- | --- | --- |
| riboflavin production | *rib*F | Bifunctional riboflavin kinase/FMN adenylyltransferase | 1 | 1 | 1 | 1 |
|  | *rib*Z | Riboflavin transporter RibZ | 16 | 16 | 16 | 15 |
| bioactive peptide | *Lux*S | S-ribosylhomocysteine lyase (EC 4.4.1.21) | 1 | 1 | 1 | 1 |
|  | *atp*C | ATP synthase epsilon chain | 1 | 1 | 1 | 1 |
| lactic acid | *pyk* | Pyruvate kinase | 1 | 1 | 1 | 1 |
|  | *ldh*D | D-lactate/D-glycerate dehydrogenase | 1 | 1 | 1 | 1 |
|  | *ldh* | L-lactate dehydrogenase | 6 | 6 | 6 | 6 |
| immune regulation | *rfb*X | Putative O-antigen transporter | 1 | 0 | 3 | 2 |
| extracellular polysaccharide | epsL | putative sugar transferase EpsL | 1 | 0 | 1 | 0 |
|  | *eps*H | Putative glycosyltransferase EpsH | 3 | 0 | 3 | 0 |
| acid-resistant | *arg*H | Argininosuccinate lyase | 1 | 1 | 1 | 1 |
|  | *dap*A | 4-hydroxy-tetrahydrodipicolinate synthase | 3 | 3 | 1 | 3 |
| bile salt resistant | *pur*D | Phosphoribosylamine--glycine ligase | 1 | 1 | 1 | 1 |
|  | *gcv*H | Glycine cleavage system H protein | 1 | 1 | 1 | 1 |


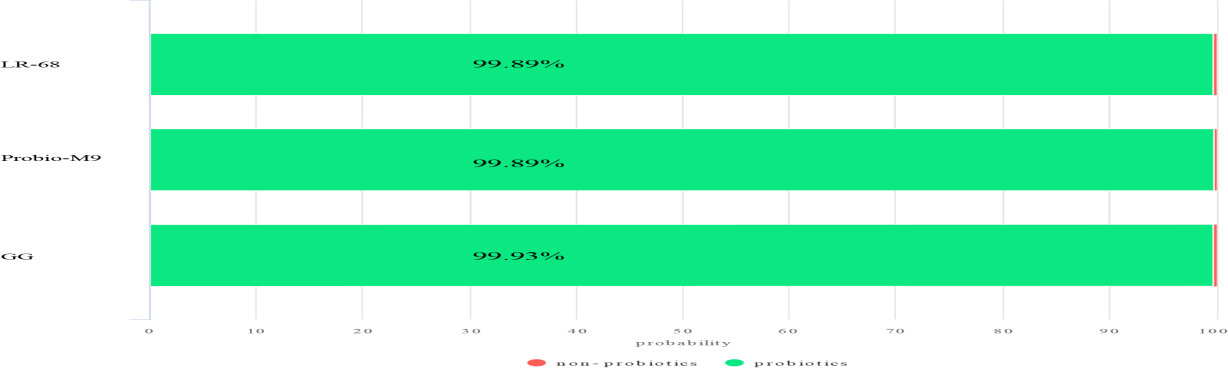


**Figure 4 iprobiotics platform prediction results**

**5、****Annotation of resistance** **genes analysis**

The Perfect and Strict algorithms in RGI 5.1.0 software were used as screening criteria to compare the genomes of *Lactobacillus rhamnosus* Glory LG12 and GG, Probio-M9, and HN001 with CARD (The Comprehensive Antibiotic Resistance Database) database to predict antibiotic resistance-related genes in the strain genome. The results showed that no potential resistance-related genes were detected in the genomes of *Lactobacillus rhamnosus* LR-109 and GG, Probio-M9, and HN001.

In addition, comparisons were made with the ResFinder database（https://cge.cbs.dtu.dk/services/ResFinder/）, which includes 2,817 resistance gene sequences on 15 major classes and 49 antibiotics. The default parameters of this database, i.e., sequence similarity >90% and sequence coverage >60%, were used as screening criteria. The results were consistent with the CARD results.

**6、****Annotation of toxicity genes analysis**

The above strains were compared with the VFDB database (Virulence Factors of Pathogenic Bacteria) and the VirulenceFinder database (https://cge.cbs.dtu.dk/services/VirulenceFinder), with sequence similarity >90% and sequence coverage >60% as screening criteria. The results showed that no potential virulence-related genes were detected in the genomes of *Lactobacillus rhamnosus* Glory LG12 and GG, Probio-M9, and HN001.

**7. conclusion**

Whole genomes of *Lactobacillus rhamnosus* Glory LG12, GG, Probio-M9 and HN001 were analysed, and the genome sizes and the number of CDSs were more consistent and genetically related among the strains. Predictive analysis of tolerance genes revealed that *Lactobacillus rhamnosus* Glory LG12, GG, Probio-M9 and HN001 contained the same genes related to thermotolerance, oxygen tolerance, acid tolerance, bile salt tolerance and cold tolerance. However, *Lactobacillus rhamnosus* Glory LG12 contained 13 unique genes (*eps*L, *rfa*B, *rfb*X, *yia*C, *dld*, *men*B, *men*E, *sau*U, *nfr*A, *xyl*A, and *nan*K) mainly associated with immunomodulation and polysaccharide synthesis. In addition, all four strains contained probiotic genes associated with riboflavin, lactic acid, bioactive peptide synthesis, and with immunomodulation, and the iprobiotics platform predicted that *Lactobacillus rhamnosus* Glory LG12 had a 99.89% probability of being a probiotic. No genes related to antibiotic resistance were detected in the genomes of *Lactobacillus rhamnosus* Glory LG12, BB-12, Probio-M8, and V9, and none of them contained any virulence genes.
